# Access, inequalities and annual health checks (AHCs) for adults living with severe mental illness in the UK: a mixed-methods systematic review

**DOI:** 10.1136/bmjopen-2024-093426

**Published:** 2025-08-04

**Authors:** Janine Owens, Rathi Ravindrarajah, Gill Norman, Elinor Hopkin, Chunhu Shi, Karina Lovell, Penny E Bee

**Affiliations:** 1 School of Nursing, Midwifery and Social work, The University of Manchester, Manchester, UK; 2 NIHR Innovation Observatory, University of Newcastle upon Tyne, Newcastle upon Tyne, UK; 3 Faculty of Biology, Medicine and Health, The University of Manchester, Manchester, UK

**Keywords:** MENTAL HEALTH, Health Services Accessibility, Delivery of Health Care, Integrated, Schizophrenia & psychotic disorders

## Abstract

**Abstract:**

**Objectives:**

Individuals living with severe mental illness (SMI) are at a significantly higher risk of mortality. This mixed-methods systematic review identifies and explores factors, including access inequalities to annual health checks (AHCs), for people living with SMI sharing protected characteristics in the UK, as identified in Core20PLUS5.

**Design:**

Mixed-methods systematic review.

**Data sources:**

MEDLINE, EMBASE, PsycINFO, CINAHL, ASSIA, Google Scholar and the grey literature were searched from 1 January 2004 to 30 January 2025.

**Eligibility criteria:**

Inclusion criteria were adults >18 years of age living with SMI. We included studies of AHCs, short health screening interventions, health promotion interventions, considering or aiming to improve uptake and/or access to screening for people living with SMI. We included mixed-methods and quantitative studies: randomised controlled trials, non-randomised controlled studies, cohort studies, cross-sectional studies and process evaluations. We also included qualitative studies.

**Data extraction and synthesis:**

Two reviewers independently assessed the evidence for inclusion using the eligibility criteria at title, abstract and at full-text screening. Quality Assessment with Diverse Studies was used for methodological quality. Analysis used Levesque’s Conceptual Framework of Access as an a priori framework and dimensions of equality from Core20PLUS5 and PROGRESS PLUS. Separate and independent quantitative and qualitative narrative syntheses and integration of the evidence from both occurred.

**Results:**

36 studies were included. Five studies applied reasonable adjustments to increase access to AHCs but lacked evaluation, controls and comparisons. 26 studies failed to discuss deprivation or ethnicity and only 6 studies discussed barriers and facilitators of access to AHCs for people of different ethnic, linguistic or cultural backgrounds. There was no evidence for interventions improving access to AHCs. Access focused primarily on dimensions of services, over abilities to access AHCs for people living with SMI.

**Conclusions:**

There are access inequalities to AHCs for people living with SMI sharing protected characteristics. Robust research is urgently needed to identify, modify and ameliorate barriers to the policy recommended AHCs.

**PROSPERO registration number:**

CRD42023437905.

**STRENGTHS AND LIMITATIONS OF THIS STUDY**

- The strength of this systematic review is that it follows Cochrane, JBI guidance and reported using the Preferred Reporting Items for Systematic Reviews and Meta-Analyses (PRISMA) to ensure the methodology was robust and systematic.
- The study did not include younger people <18 years of age living with severe mental illness; therefore, insights are limited to adults.
- Using the conceptualisation of access by Levesque *et al* is a strength because it provides a structured *a priori* framework to identify evidence of the dimensions and abilities of access.
- The review focuses entirely on the UK because of its configuration of service provision, which may be a limitation.
- The lack of evidence for interventions which enable access to annual health checks is a limitation of the review.

## Introduction

 The National Health Service (NHS) long-term plan defines severe mental illnesses (SMI) as a diagnosis of schizophrenia, bipolar affective disorder and other psychoses (and other patients on lithium therapy within the preceding 6 months).^1^

In England alone, approximately 587 000 people live with SMI, with an overall estimate of 2% of the population living with SMI in the UK.^2^ Data collected by general practitioner (GP) practices are imprecise because various influential factors including changes in prevalence of the condition within the population; demographic changes, such as an ageing population; improvements in case finding by practices, different ways of recording and a lack of consistency with codes.^3^,^5^ Furthermore, it excludes people experiencing mental health problems in hospitals, prisons, sheltered housing and those who are homeless.^6^ Individuals living with SMI are at a significantly higher risk of mortality, with evidence suggesting a reduced life expectancy of up to 25 years compared with the rest of the population.^7^ Around two-thirds of these deaths result from comorbidities.^8^

Individuals living with SMI are at higher risk of diabetes, cardiovascular disease, obesity, cancer, respiratory diseases, liver abnormalities and obesity.^9 10^ There are a range of modifiable and unmodifiable risk factors for people living with SMI such as smoking, poor diet, obesity and substance abuse.^11^ The adverse effects of psychotropic medicines increase risk factors for metabolic syndrome, thyroid disease, cardiovascular disease, respiratory illness, weight gain and type-2 diabetes.^12^,^14^ People diagnosed as living with SMI also have increased use of Accident and Emergency services (A&E) and hospital admissions for both physical and mental health conditions.^15^,^17^ Despite the extensive evidence, individuals living with SMI are less likely to undergo general health screening in primary care.^18^ In the UK, a range of settings manage individuals living with SMI (eg, primary and secondary care). Primary care also provides annual physical health checks for individuals identified as living with SMI.^19 20^

In 2004, the UK’s NHS implemented the Quality and Outcomes Framework (QOF) for all four countries (England, Wales, Scotland and Northern Ireland), with the objective of improving the quality of care given to patients. The QOF was based on several indicators across a range of key areas of clinical care and public health. At the time, it was the world’s largest pay-for-performance healthcare scheme.^21^ Scotland abolished the QOF in 2016 to reduce the bureaucratic burden on GPs and to liberate their time for patients. This was associated with reductions in recorded quality of care for most performance indicators, but no significant differences in physical and mental health for people with SMI compared with England.^22^ Furthermore, despite the introduction of the QOF incentive of annual physical health checks for adults living with SMI 8 years previously, the 2012 National Audit of Schizophrenia for England and Wales reported that less than one-third of patients received a completed cardiometabolic risk assessment, and even if assessed, many people with identified risks received no intervention.^23^ Despite sustained policy recommendations, progress has been slow in the last decade.

Limited evidence suggests that there are disparities in the ways provision of annual health checks (AHCs) occurs.^24^ Reasons for these disparities vary. For example, a lack of continuity of care, patient withdrawal from care, negative practitioner attitudes towards patients and a lack of awareness about the differing starting points and support needs of people living with SMI.^25 26^ In America, more focused interventions include a nurse-led case management approach to improving access to AHCs.^27^ A small-scale study of the use of the health improvement profile by non-government organisations in Australia found an improvement in access to AHCs for people living with SMI.^28^ While a Cochrane review on collaborative care for SMI found low certainty of evidence that collaborative care was more effective than standard care in improving the physical and mental health of people living with SMI.^29^

The NHS Long Term Plan aimed to improve access to physical health checks, with a proposed 390 000 health checks for people living with SMI in England by 2023/2024.^30^ In 2021, the NHS introduced Core20PLUS5, of which one explicit objective was to reduce health inequalities and ensure AHCs for 60% of individuals living with SMI, as one of the five clinical areas identified for improvement.^23^ ‘PLUS’ population groups include groups sharing protected characteristics as defined by the Equality Act 2010 and other socially marginalised groups; such as travellers, sex workers, people experiencing homelessness and people with drug and alcohol dependence. The optimal mechanisms by which to achieve these improvements are not specified. Even though the central tenet of the UK Core20PLUS5 framework is to reduce inequalities through improved healthcare access, it fails to identify how this may occur or what examples of good practice may already exist for marginalised groups.

The paucity of evidence about AHCs means that this review aims to identify and explore their accessibility for people living with SMI in the UK, as a starting point for proposing evidence-informed solutions and theories of change and to examine whether the evidence on SMI and access to AHCs represents marginalised groups identified in Core20PLUS5.

## Methods

The range and type of evidence needed to answer the aims and research questions meant that this would be a mixed-methods systematic review. The review follows the updated Joanna Briggs Institute (JBI) methodological guidance.^31 32^ It uses the Preferred Reporting Items for Systematic Reviews and Meta-Analyses (PRISMA).^33^ The review protocol was registered on 21 June 2023 on the International Prospective Register of Systematic Reviews (PROSPERO), registration number CRD42023437905. The protocol was modified after the review began to increase clarity of wording for the outcomes. The methods aligned with a mixed-method systematic review and the title was altered as a result.

This review employs a UK focus because of its configuration of service provision, and this may be a limitation. However, the depth of exploration employed meant that some aspects of the review could transfer into international settings.

Specifically, the paper addresses the following questions:

What are the barriers and facilitators of access to AHCs for people living with SMI?

What features, actions, interventions or models of service provision improve uptake and access to AHCs for people living with SMI?

To what degree does the evidence on SMI and access to AHCs represent marginalised groups as identified in Core20PLUS?

What are the barriers and enablers of access to AHCs for people living with SMI from marginalised groups, as identified in Core20PLUS?

### Patient and public involvement

This was a systematic review of access to AHCs for people living with SMI. There was minimal funding and tight time scales. This meant it was not appropriate to ask a diversity of people with lived experience to employ their time for no recompense; there were limited resources to develop individuals to gain insight into research methods, critically appraise existing evidence or develop a diverse group and the relationships of trust required for effective working. Therefore, it was not possible to involve people with lived experience in the design, conduct, reporting or dissemination, which is a limitation of this systematic review.

### Search

We searched the following databases: MEDLINE (OVID), EMBASE (OVID), PsycINFO (OVID), CINAHL (EBSCO), Applied Social Science Index & Abstracts [ASSIA] (via ProQuest), the first 10 pages of Google Scholar and the grey literature (see online supplemental table 1). Retrieved articles were exported to an EndNote library and duplicates were removed. We also used backward and forward searching of retrieved records, identified through initial searches meeting eligibility criteria, to identify any additional relevant literature for inclusion. Forward searching of references within retrieved records, cited in an article after its publication, enabled finding new theoretical developments about AHCs for people living with SMI and consideration of any other methodologies employed. Second generation forward searching (examination of sources cited by the references used in an initial article) enabled the researchers to search for inconsistencies. Backward searching meant identifying and examining the references or works applying to this study and enabled learning around the development of AHCs for people living with SMI, while identifying experts in the area. We limited searches to adults and publications written in English. Searching occurred from the introduction of the QOF from 1 January 2004 up to 30 January 2025.

### Search terms employed

The search strategy employed the following facets: SMI; health checks; access. The terms were designed with the assistance of the University librarian. It also used Medical Subject Headings (MeSH) terms to expand the search. The full search strategies for MEDLINE, EMBASE, PsycINFO, CINAHL, Applied Social Science Index & Abstracts [ASSIA] and the first 10 pages of Google Scholar are in online supplemental table 2. We amended terms depending on the databases used.

### Eligibility criteria

We ran preliminary searches to develop the eligibility criteria. Specific inclusion/exclusion criteria are defined by population, interventions, comparators and outcomes (PICO).

### Population

We included studies of adults aged 18 years and above with a current diagnosis of SMI as defined by the National Institute of Mental Health and which discussed access to health checks. People with and without comorbidities were eligible. We included studies of both adults and adolescents, provided that at least 75% of participants were adults, or studies with separate outcome data for adults. Where studies included both people with and without diagnosed SMI, we included them, provided that at least 75% of participants had a diagnosed SMI or separate outcome data was available for those living with SMI. We excluded studies of staff perceptions and studies testing data collection tools, if they did not focus on access to AHCs for people living with SMI.

We included people identified as living with SMI as defined within the (NHS) long-term plan; as a diagnosis of schizophrenia, bipolar affective disorder, and other psychoses (and other patients on lithium therapy within the preceding 6 months).^1^ We did not include studies focusing only on dementia, personality disorders and intellectual disability. However, we included people with intellectual disabilities and people with a diagnosis of a personality disorder living with SMI. We excluded studies about SMI, which included adults with depression without separate outcome data.

### Interventions

We included studies of AHCs, short health screening interventions, health promotion interventions or reasonable adjustments, which considered or aimed to improve uptake and/or access to screening for people living with SMI. We excluded studies of health checks focusing entirely on a particular physical illness (eg, an annual asthma or diabetes review) or reviews only of the primary diagnosed SMI (eg, annual reviews of medication for schizophrenia or bipolar disorder), unless they discussed other aspects related to increasing access to a health check. Checks took place in any healthcare setting or in the community.

### Comparator

We included studies with no comparator, care as usual, alternative approaches to provision of health checks or alternative approaches to adjustments or methods of increasing access to health checks.

### Outcomes

We considered two outcome domains in this review (a) measures of access or interventions designed to improve access for people sharing protected characteristics living with SMI and (b) accessibility of health checks for people sharing protected characteristics living with SMI. We used the model of access developed by Levesque *et al*^34^ as an *a priori* framework to code how each study measured dimensions of accessibility.

Our primary outcome is evidence of access to health checks for people sharing protected characteristics living with SMI.

Our secondary outcomes are:

- Evidence of changes in uptake of AHCs for people sharing protected characteristics living with SMI
- Dimensions of access to health checks and abilities of people living with SMI to access AHCs.
- The representation of protected characteristics, as identified in Core20PLUS, for people living with SMI.

### Study designs

We included mixed-methods and quantitative studies with the following designs: randomised controlled trials, non-randomised controlled studies, cohort studies, cross-sectional studies and process evaluations. We also included qualitative studies. We did not include case studies, reports, opinion pieces, editorials, systematic and scoping reviews, but instead used reviews to identify primary studies.

### Inclusion screening

To check for consistency, two reviewers (JO and RR) screened an initial 100 references. Discussion resolved queries with the other reviewers (GN, KL, CS and PEB). The two reviewers then independently assessed the evidence for inclusion using the eligibility criteria at both title and abstract and at full-text screening. Discussion and reaching consensus with the wider review group resolved any disagreements.

### Extraction and tabulation of data

We developed a bespoke data extraction form with separate elements for quantitative and qualitative data. Tabulation of characteristics of the evidence included (a) author, date, peer reviewed or grey literature, (b) UK country, (c) research design and methods employed, (d) healthcare setting, (e) sample size, (f) % female, (g) age range, (h) SMI diagnosis, (i) diagnosed comorbidities, (j) ethnicity or race, (k) accommodation arrangements, (l) person seen, (m) type of health check, (n) hospital admissions, (o) barriers to and facilitators of access and (p) increasing access to AHCs. We piloted the form using two researchers (JO and RR) on a small set of studies representing the range of included study designs. Subsequently, one researcher (JO) extracted the data and another (RR) checked it.

We ran PROGRESS-PLUS on the emergent data to map the evidence.^35^ PROGRESS-PLUS is a tool to guide, consider and use as a lens to identify equity in research. The acronym refers to Place of residence, Race/ethnicity/culture/language, Occupation, Gender/sex, Religion, Education, Socioeconomic status and Social capital. We combined PROGRESS-PLUS with the protected characteristics in the Equalities Act 2010, which Core20PLUS5 focuses on. This produced the following characteristics: age, disability, education, gender and sex (including gender identity), occupation, place of residence, pregnancy/maternity, ethnicity, race, religion, sexual orientation, marital status, social capital, socioeconomic status, people experiencing homelessness, trafficking, street sex workers, refugees, people in contact with the criminal justice system.

We also used Levesque’s five dimensions of accessibility and abilities of people to interact with the dimensions as an a priori framework to identify evidence of the dimensions and abilities of access. Access is a multidimensional concept and Levesque’s model conceptualises five dimensions of accessibility (Approachability; Acceptability; Availability and accommodation; Affordability; Appropriateness) and five corresponding abilities of populations (Ability to perceive; Ability to seek; Ability to reach; Ability to pay; Ability to engage) to generate access.^34^ Within this framework, marginalisation is viewed as a potential and specific barrier to access. This review uses Levesque’s dimensions and abilities of access as an *a priori* framework to summarise the evidence and identify barriers to and facilitators of access.

For quantitative outcomes, we aimed to extract data to permit the calculation of relative risks with 95% CIs or mean or standard mean differences with 95% CIs where appropriate.

### Assessment of methodological quality

Three researchers (JO, RR and EH) used the Quality Assessment with Diverse Studies (QuADS) tool to appraise the methodological quality of included studies.^36^ The tool assesses various important methodological aspects of studies, such as the underlying theory, defined objectives, appropriateness and rigour of the design, data collection methods and analytical methods. It consists of 13 evaluative indicators (see online supplemental table 4: QuADS questions for evaluative indicators and quality analysis), each rated on a 4-point Likert scale ranging from 0 (not at all) to 3 (complete), allowing researchers to determine the extent to which each criterion is met. To ensure consistency within the quality assessment, two researchers (RR and EH) carried out an initial pilot on 10% of the sample. They independently evaluated the quality of the studies, and one researcher (JO) resolved any discrepancies through discussion. For the remainder of the papers, discussion within the review team resolved any conflicts of agreement.

### Analysis and synthesis

We aimed to calculate effect estimates (with 95% CIs) for the quantitative data but were unable to do so in all cases and therefore report available data, however limited.

There were different questions for the review focusing on different aspects of the same phenomenon in this mixed-methods systematic review; therefore, the synthesis took a convergent segregated approach. This consisted of conducting separate and independent quantitative narrative synthesis and qualitative narrative synthesis, followed by integration of the evidence derived from both syntheses.^31 32^ Integrating the qualitative and quantitative studies synthesises findings and offers a greater depth of understanding around access to health checks for people living with SMI.

## Results

**Figure 1:**
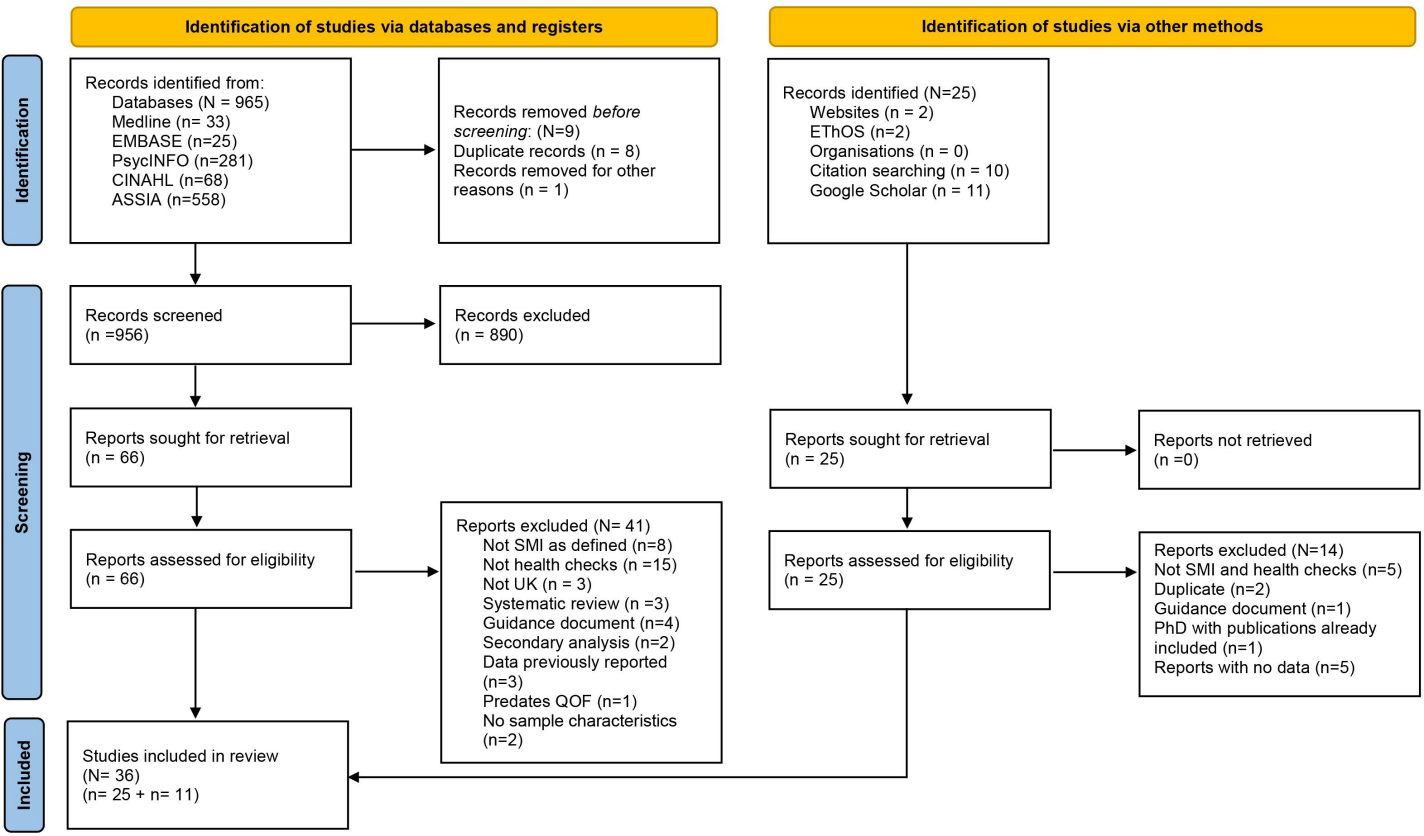
PRISMA flow chart. Adapted from Page *et al.*^33^ PRISMA, Preferred Reporting Items for Systematic Reviews and Meta-Analyses; QOF, Quality and Outcomes Framework

The PRISMA flow chart (figure 1) illustrates the search results. Out of 981 records and after application of the eligibility criteria, 36 studies were included in this review.

### Characteristics of studies

Online supplemental table 3 gives the characteristics of the 36 studies included in this review.

Research designs for the 36 papers comprised 14 (38.89%) cross-sectional; 12 (33.34%) clinical audit; 4 (11.11%) qualitative; 4 (11.11%) service evaluation and 2 (5.56%) mixed-methods. The majority of studies; 28 (77.78%), occurred in England.

In terms of range of healthcare settings, 24 (66.67%) studies occurred in primary care, 8 (22.22%) in secondary care and 4 (11.11%) were a mixture of primary and secondary care.

The level of diversity in the study designs meant sample sizes ranged from 9 to 346 551 with a median of 227. The age range for studies was 16–93 years. Although, eight studies omitted to give ages or ranges.^37^,^44^ 19 studies (52.78%) failed to acknowledge ethnicity, 17 (47.22%) attempted to define ethnicity in multiple ways.^4345^,^60^ For example, two studies combined minority ethnic groups (eg, black, minority ethnic groups)^46 56^, possibly because of small sample sizes and one study classed ethnicity as non-white,^58^ possibly because it reported at practice level and not patient level.

### Analysis and effect estimates

The variable quality of identified evidence meant it was not possible to calculate effect estimates (with 95% CI) for quantitative data because of the limited evidence about AHCs and marginalised groups living with SMI. This reflected the infancy of the research in AHCs for people living with SMI, diversity in the populations and research designs. Opportunities for pooling data for meta-analysis were explored, but the evidence precluded conducting a meta-analysis. Therefore, a narrative analysis occurred reflecting on the barriers and facilitators of access and a priori framework of access and the evidence on equity and protected characteristics.

### Quality of studies

The variety of research designs in the evidence meant the quality of the studies was assessed using the Quality Appraisal for Diverse Studies (QuADS)^36^ (online supplemental table 4). Quality assessment of the included studies using QuADS gives a complete overview, alongside the questions and coding criteria.

Methodological quality varied. Some studies demonstrated explicit theoretical or conceptual frameworks, clear descriptions of the research setting and appropriate sampling methods; others lacked these crucial elements. The choice and justification of data collection tools and analytic methods varied, with some studies offering detailed justification and explanation, while others offered either rudimentary or no accounts. Seven studies (19.44%) actively engaged stakeholders in the research design.^43 45 51 55 57 61 62^ In contrast, 29 studies (80.56%) omitted to actively include participants in the research design or other aspects of the study. 72.22% of studies were based either on routine primary care records of patients or routinely collected data and, therefore, the data may have been subject to recording bias. A further 11.11% of studies were qualitative and may have been subject to recruitment bias.

### The barriers and facilitators of access to AHCs for people living with SMI in the UK

Working between onlinesupplemental tables 3  5, we can infer that all studies identified that appointments were issued for people to attend AHCs, but that the process of issuing appointments could be ‘unreliable’.^55^ This may be attributed to differing processes from practice to practice and case mix for different areas. The healthcare access barriers model (HCAB)^63^ targets HCABs that are measurable, modifiable and can be the subject of public health and clinical interventions. Box 1 presents the barriers to accessing AHCs using HCAB.

Box 1 **Barriers to access using the healthcare access barriers model^63^**

**Structural barriers**

Quality and Outcomes Framework (QOF) indicators: used variably and omitted people with schizophrenia,^85^ if patients ‘did not attend for their annual health check (AHC) they were not followed up’,^49^ ‘attending to QOF targets contributed to a disease focus on risk and recording’,^86^ a ‘reduction in aspects of care that were non-incentivised’^53 58^, an increase in hospital admission rate of 0.19% (95% CI 0.10% to 0.28%) or 0.007 patients (95% CI 0.003 to 0.01)^58^, ‘risk of admission to hospital for ambulatory care and sensitive conditions was 23% lower (HR 0.77, 95% CI 0.60 to 0.99) for patients living with severe mental illness (SMI)’.^61^

Service provision: ‘poor communication between primary and secondary care and the patient’^3840 45 49 50 55 57 60^,^62 65 79 86 87^. ‘lack of basic equipment’, ‘understanding about monitoring’, ‘poor electronic coding’ and ‘unreliable systems for sharing patient results’^40 50 88^, ‘lack of staff motivation to implement health checks’,^60^ a lack of ‘baseline data making follow-up for AHCs difficult’.^38^ A ‘need for staff training in SMI and multimorbidity’^3840 45 51 59^,^61^, and ‘heavy staff workloads preventing screening’.^65^ Provision and distribution of clinics’^46 51^, ‘proximity of services’,^58^ ‘availability of transport’^51 65 79^, ‘level of mobility’.^79^ ‘Seeing a GP regularly enhances access to care (p<0.11)’.^41^

**Financial barriers**

Deprivation: affects ability to travel.^47^

**Cognitive barriers**

Mental health: ‘mental health status of the person living with SMI’^40 57 70 88^.

Comorbid conditions: ‘prioritising management of mental illness and avoiding AHCs, potentially to the detriment of their diabetes’,^70^ comorbid condition made people living with SMI ‘2.2 times more likely to attend an AHC compared with those with SMI alone (OR 52.20, 95% CI 51.13 to 3.62)’.^71^

Trust: developing relationships of ‘trust between the person living with SMI and practitioner, nurse, community mental health team or peer supporter’.^57 65 68 79^

Support: to identify the link between mental and physical health and the link with comorbidity in order to pursue AHCs^48 60 70 71 79^, improves ability to perceive the need for an AHC.^57 68^ Ensuring those who knew the person the best are included to support people with learning disabilities living with SMI to attend the AHC appointment.^39^

### Features, actions, interventions or models of service provision improving uptake and access to AHCs for people living with SMI

We could not assess the effectiveness of interventions to improve uptake of access to health checks for people living with SMI. This was because the five studies in the review did not contain control groups or comparators, did not evaluate and were therefore mostly descriptive or feasibility studies describing improving access to health checks.

### Improving access to AHCs

Online supplemental table 3 contains five studies^40 42 57 64 65^ describing ways of improving access to health checks for people living with SMI. Two focused on ‘upskilling staff’ by focusing on increasing knowledge of the physical health risks for people living with SMI.^42 64^ The five studies were all descriptive with no comparative evaluation. Examples included:

Setting up a specialised clinic to carry out AHCs and screening for people living with SMI and work with community mental health teams to integrate care.^65^ The study did observe the ‘resource challenges with a specialised clinic and difficulties with trying to integrate physical and mental healthcare’.

Using physician associates (PAs) to carry out treatment and support people living with SMI in their homes, they worked closely with people’s GPs to develop treatment plans for 71 hard-to-reach people living with SMI.^40^ The study demonstrated the integration of PAs into a community mental health multidisciplinary team to support patients living with SMI to access screening and other interventions to improve their health.

Employing a peer coach with ‘lived experience of SMI’^57^; people living with SMI attending for health checks were ‘encouraged to set own physical and MH goals which they discussed with the peer’. The control for health was placed in the hands of the individual living with SMI and they were supported to make choices and decisions. Peer support workers have been used with varying degrees of enthusiasm in mental health services. In Ireland, it is part of the national mental health strategy, while in other countries, local or regional services are left to decide whether and how to implement it. West Sussex began peer support in 2019 and the main struggles have been limited resourcing, valuing the ethos of peer support, and convincing local commissioners because of lack of evidence.^66^ Support services staffed by people with lived experience of mental health difficulties are currently being implemented in formal healthcare settings and have begun to spread across a number of European countries,^67^ however, comparative evaluation is still lacking.

Building ‘trusting relationships’ with staff, which, although lacking in evaluation, appeared to ‘enable people living with SMI to access health checks and provided support’ for further services if needed.^40 57 65^

### The evidence on SMI and access to AHCs representing marginalised groups as identified in Core20PLUS

AHCs for people with SMI using PROGRESS Plus^35^ and Core20^23^ denote the spread of characteristics included in the studies. 27 studies did not mention the Index of Multiple Deprivation (IMD), or deprivation as a barrier to access for people living with SMI. Six studies mentioned the IMD when collecting characteristics of the sample but did not discuss further.^44 47 56 60 61 68^ Three studies discussed people with SMI living in the most deprived quintiles.^48 52 69^ (online supplemental table 6)

In terms of race, culture, ethnicity and language, 21 studies (58.33%) failed to acknowledge ethnicity, 12 (33.33%) collected ethnicity as part of routine data but then did not discuss further.

Out of the 36 studies, only 2 noted occupation.^46 51^ Only 10% of people living with SMI were employed and that this may constitute a barrier to accessing AHCs because of ‘trying to arrange appointments and missing work’.^46^

Eight studies failed to include the characteristics of gender or sex (including gender identity),^38 39 42 43 62 64 70 71^ and 20 studies mentioned the characteristic but then did not discuss further. One study found no difference between male and female groups from different ethnicities regarding screening.^52^ Seven studies^44 46 48 50 55 68 69^discussed sex and gender in various ways, but all were observational rather than derived from comparative evaluation.

No studies included sexual orientation, religion or social capital, and only one study noted education in their sample characteristics but did not discuss further.^47^ Socioeconomic status was in the sample characteristics for only two studies, but the authors did not discuss its importance in accessing health checks.^41 58^

Age was discussed as a sample characteristic in 21 studies, but its significance for accessing AHCs failed to be discussed further. Six studies omitted age in its entirety.^38^,^4042 43 70^ The remaining nine studies were contradictory and subjective.

The last two characteristics were pregnancy/maternity where only one study found that ‘9% of women under 45 years living with SMI had received pregnancy/contraception advice during health checks’,^49^ and lastly, disability where 32 out of 36 studies failed to recognise that living with SMI was classed as a disability because it was long-term and affected daily life.

Despite extensive searching, we could find no evidence about access to AHCs for people living with SMI and experiencing homelessness, in contact with the criminal justice system, refugees and asylum seekers, travellers, trafficked people and sex workers, and nothing about marital or relationship status.

### Barriers and enablers of access to AHCs for people living with SMI from marginalised groups, as identified in Core20PLUS

Some studies found barriers to access for people of different ethnicities played a minor part in access to AHCs by ethnicity.^50 54 60^ Only six (16.67%) studies discussed barriers and facilitators of access to AHCs for people of different ethnic, linguistic or cultural backgrounds.^43 48 52 55 57 68^

People with a South Asian background living with SMI had a ‘reduced mortality rate’ and this was subjectively attributed to increased health checks for type 2 diabetes.^68^

Participation in screening by adults of an ethnic minority background living with SMI was ‘particularly low’,^48^ with no reason offered.

People living with SMI of black (vs white) ethnicity were more likely to always have complete screening in the 2014–2018 period only (OR 1.39; 95% CI 1.19 to 1.62); attributed to the ‘QOF indicators and more people being invited for screening’.^55^

‘Discriminatory attitudes of staff created barriers to access’^52^

Language was a barrier to ‘carrying out health checks for people living with SMI if no translator was available’^57^

‘Lack of cultural competence of healthcare staff was a barrier for people living with SMI from different ethnic and cultural backgrounds’.^72^

The impact of deprivation and its link to disabling environments was highlighted with one study using Core20PLUS as a framework finding participation in health checks was lowest among people living in the most deprived quintiles both for people living with and without SMI (bowel: 36.17% and 47.86%, respectively; breast: 40.23% vs 50.36%; cervical: 61.47% vs 64.59%),^48^ and another finding a higher than average prevalence of psychosis, ‘linked to deprivation and ethnicity characteristics of the area’.^52^ The lack of explanation as to the ways deprivation and ethnicity interact in these studies limits further interpretation. The impact of deprivation indicated that people living in the most ‘deprived areas (quintiles Q4 and Q5) were less likely to attend an NHS Health Check than those in the most affluent quintile’,^48^ people living with schizophrenia were more likely to live in a deprived quintile^69^ and there was a higher than average prevalence of psychosis, linked to ‘deprivation and the ethnicity characteristics of an area’.^52^ Reasons why people with SMI lived in more deprived quintiles were not explored.

## Discussion

This systematic review produced 36 studies for further analysis. The evidence around access to AHCs for people living with SMI was frequently contradictory, and this is possibly because of the different research designs and methods employed. Furthermore, there was little robust evidence about people sharing protected characteristics living with SMI. These are all limitations of this review.

Barriers and facilitators of access emerged from using QOF indicators, which were found to increase in-patient admissions for one study,^58^ but reduce them in another.^61^ This queried how useful a measure QOF indicators were, but it also implies that case mix for different practices may be responsible for these contrasting results. The majority of studies failed to include the perceptions of people living with SMI to improve services. There was some evidence of communication failures between primary and secondary care, staff lacking in cultural competence, a lack of consideration of difference between people living with SMI and a focus on conceptualising service uptake as evidence of access. Facilitators of access to AHCs for people living with SMI revolved around relationships of trust between people living with SMI and healthcare professionals.

25 out of 36 studies relied on clinical records and routinely collected data, which although useful in contributing towards understanding inequalities, merely measures whether individuals use a screening or health check service and fails to fully understand the complexities of access. While mental health status can sometimes be a reason for non-attendance at AHCs, the concept of reasonable adjustments was only considered in six studies out of 36 to support and enable people living with SMI to access AHCs.

Despite the valuable perspectives that patients, carers and the public can bring to research,^66^ over 80% of studies failed to meaningfully include participants in the design and process of the research. Although this may be related to the design of the studies, 14 (38.89%) were cross-sectional; 12 (33.34%) were clinical audit and 4 (11.11%) were service evaluation. Nonetheless, the lack of inclusion of patients living with SMI about health checks may lead to services being practitioner-led and focused, and this itself could be a barrier of access to AHCs. Without the input of people with experience of living with SMI as to what may facilitate access, then services are unlikely to move forwards. Moreover, improving understandings from the perspective of people living with SMI about barriers to accessing health checks could improve access.

The evidence reported focused more on structural factors and QOF indicators were viewed as a barrier and facilitator to AHCs. Criticisms of the QOF indicators are that they are based on clinical guidelines and process indicators.^73^ Therefore, they do not consider person-centred care for people needing more individualised support, nor do they consider continuity of care which involves prevention, health promotion, reduction of hospital admissions and premature mortality.^74^,^75 76^Evidence within this review around the QOF indicators appears contradictory, indicating that it is both a barrier to and facilitator of access to AHCs. However, for the two conflicting studies on hospital admission,^58 61^ differences could relate to documentation of QOFs postadmission or patients who may not regularly seek primary care and are more likely to seek A&E care.^77 78^ They could also emerge from unobserved factors contributing to ‘a (non-causal) positive association, such as care plans being triggered by patients attending their GP for problems that will eventually need hospital care’ (p.6).^61^ This questions the utility of the indicators as a measure. Furthermore, if the QOF indicators are utilised as a tick-box exercise, this can contribute to a ‘reduction in aspects of care that are non-incentivised’.^53^ If people living with SMI attend an AHC because ‘they are invited, rather than understand its necessity’,^79^ then this fulfils the measure of uptake but it also has implications for health literacy, choice and decision-making, and a potential failure to improve health outcomes.

Service provision barriers related to communication between primary and secondary care. Previous research argues that timely and accurate communication between primary and secondary care is essential for delivering high-quality patient care.^80 81^ This review suggests that changes to the way that services are delivered and integrated, especially those that strengthen links between primary and secondary care, potentially improve access to health checks and quality care.^68 69 71^ The British Medical Association supports this position, simultaneously arguing that post-COVID-19 barriers include “[…] high workload, the need to adapt physical spaces to prevent the spread of infection, lack of joined up IT, historic workforce shortages and a lack of consistent communication and trust between different parts of the health system”^82^(p.2). The review also identifies a lack of cultural competence of healthcare staff as a barrier for people living with SMI from different ethnic and cultural backgrounds.^51^ Cultural incompetence can lead to poor quality care, negative health consequences and poor health outcomes.^83^ This can act as a barrier to accessing AHCs and receiving follow-on care.

There was a lack of routine consideration when accounting for differences in need and experience. Over half of the studies failed to include ethnicity, and the studies including this characteristic frequently failed to identify its importance or differentiate between groups. This may be wholly related to sample size and the study question. However, combining black and ethnic minority groups presumes that they all have a shared experience and inhabit similar socioeconomic contexts, which can be a barrier to accessing AHCs. Likewise, not exhibiting any diversity in the sample may be problematic for the evidence base because it fails to identify evidence of barriers and facilitators of access. People are shaped by their membership in multiple social categories, such as race, gender, class, sexuality and ability, which is known as intersectionality. Research providing more intersectional accounts offers the potential to increase understandings about inequalities in access to AHCs for groups sharing protected characteristics.

A few unsupported small studies indicate positive impacts with the involvement of key or peer support workers and Community Mental Health Teams (CMHTs) who built trusting and enabling relationships with people living with SMI. These relationships may be key facilitators of access to AHCs. One recent systematic review in nursing identifies that outside psychotherapy there is little evidence about developing dyadic, mutual, enabling and professional relationships.^84^ However, the paucity of evaluated studies limits identification of the key factors involved in developing relationships with people living with SMI to enable access to health checks.

### Strengths and limitations of the review

The strengths of this mixed-methods systematic review are that it was rigorously planned, used PICO to formulate the research questions and applied a strict eligibility criteria for selecting primary studies for the review. Every stage was predefined and made publically available through PROSPERO, before the study commenced, and the supplemental tables after commencement, making it transparent and reproducible. The review was reported using PRISMA to ensure the methodology was robust and systematic. The results were analysed by taking a convergent segregated approach. One limitation here was the quality of the evidence. The lack of robust research designs, relative risk of bias and the disparate evidence meant confirmability and adopting the Grading of Recommendations, Assessment, Development and Evaluations framework was not possible. Conclusions were therefore based on narrative synthesis. Between-study heterogeneity was not assessed quantitatively because a meta-analysis could not be conducted. The lack of randomised controlled trials in the evidence meant risk of bias could not be calculated.

The primary sources were collected from five main databases, Google Scholar, the grey literature, independent websites and, in addition, a manual search from the reference lists of retrieved papers and review articles was also performed. This was exhaustive. Using multiple databases ensured a comprehensive search that encompassed diverse perspectives and disciplines relevant to access to AHCs for people living with SMI. Disagreement among reviewers as to which papers to include was resolved through discussion with the other authors to reach agreement and assisted with reducing the risk of selection bias. Methodological quality of the studies varied and there was a lack of robust designs, little formal evaluation and no evidence for interventions improving access to AHCs. This meant we could not fully answer one of our review questions, which is a limitation of this review. Another limitation is that we chose to focus solely on the UK because of the differences in service configuration in other countries; therefore, we may have missed useful evidence. During analysis, even though we applied Levesque’s dimensions and abilities of access as an a priori framework to the evidence, we experienced challenges because of the difficulty in categorising certain questions or data into a specific dimension or ability of access. This is because some constructs contain similar elements and a degree of overlap occurred. This is a limitation of the work and to reduce the level of subjectivity, engaging in lengthy discussions ensured researchers reached the most appropriate decision and fit for the evidence.

## Conclusions

This review produced variable and often contradictory evidence around barriers to and facilitators of access for people living with SMI. QOF indicators were both a barrier to and a facilitator of access. Other barriers to accessing AHCs for people living with SMI relate to a lack of robust research enquiry, cultural competence, integrated care and a limited understanding of the barriers in daily living for people living with SMI. These factors also led to failings in taking a whole person perspective. The small amount of research involving the development of trusting relationships appears to facilitate access for people living with SMI, but the evidence relies on small qualitative studies and is weak. Developing pilot studies with key workers to support people living with SMI to access AHCs may be a future study. Accessing care mostly revolves around dimensions of service provision from a clinical perspective. This retains an outdated model and fails to consider the person’s journey through care. A further study to follow the journey through care for people sharing protected characteristics living with SMI, using an integrated, meaningful, evaluative framework, coproduced with all stakeholders, may identify points at which people may be facilitated to access AHCs and obtain referrals to secondary care.

## Supplementary material

10.1136/bmjopen-2024-093426 online supplemental file 1

10.1136/bmjopen-2024-093426 online supplemental file 2

10.1136/bmjopen-2024-093426 online supplemental file 3

10.1136/bmjopen-2024-093426 online supplemental file 4

10.1136/bmjopen-2024-093426 online supplemental file 5

10.1136/bmjopen-2024-093426 online supplemental file 6

## Data Availability

All data relevant to the study are included in the article or uploaded as supplementary information.
