# Supplementary material for: Access, inequalities and annual health checks (AHCs) for adults living with severe mental illness in the UK: a mixed-methods systematic review
Source: BMJ Open. 2025 Aug 4;15(8):e093426. doi: 10.1136/bmjopen-2024-093426 (PMC12323543; doi:10.1136/bmjopen-2024-093426)
Supplement: online supplemental file 1 [file bmjopen-15-8-s001.docx]

**Supplemental Table 1**. Grey Literature

| **Name** | **Website** |
| --- | --- |
| British Institute of Learning Disabilities (BILD) | [www.bild.org.uk](about:blank) |
| IHaL - The Learning Disabilities Public Health Observatory at Lancaster | [www.ndti.org.uk/our-work/our-projects/peoples-health/improving-health-and-lives-ihal](about:blank) |
| Mind | [www.mind.org.uk/](about:blank) |
| Rethink Mental Illness | [Resources and reports (rethink.org)](about:blank) |
| Mental Health Foundation | <https://www.mentalhealth.org.uk/england/our-work/research> |
| The McPin Foundation | <https://mcpin.org/about-mcpin/> |
| Royal College of Psychiatrists | <https://www.rcpsych.ac.uk/> |
| Centre for Mental Health | <https://www.centreformentalhealth.org.uk/> |
| NIHR research and evidence | <https://www.nihr.ac.uk/> |
| National Institute for Health and Care Excellence (NICE) | <https://www.nice.org.uk/> |
| Agency for Healthcare Research and Quality (AHRQ) | <https://www.ahrq.gov/> |
| Social Care Institute for Excellence (SCIE) | <https://www.scie.org.uk/> |
| EThOS British Library e-theses online service | <https://ethos.bl.uk/> |

Search Terms employed:

Severe Mental Illness, SMI, Access to health checks, Access to health checks AND SMI OR Severe Mental Illness, Annual Health Checks AND Severe Mental Illness, Psychosis AND health checks, Schizophrenia AND health checks, bipolar disorder AND health checks

Dates searched: 1.01.2004-30.01.2025

Population: Adults > 18years of age

Geographical area: UK only
