# Supplementary material for: Access, inequalities and annual health checks (AHCs) for adults living with severe mental illness in the UK: a mixed-methods systematic review
Source: BMJ Open. 2025 Aug 4;15(8):e093426. doi: 10.1136/bmjopen-2024-093426 (PMC12323543; doi:10.1136/bmjopen-2024-093426)
Supplement: online supplemental file 2 [file bmjopen-15-8-s002.docx]

**Supplemental Tables 2.**

MEDLINE search strategy

| Boolean operators | **1** | AND  2 |
| --- | --- | --- |
| MEDLINE MeSH® Search Terms and Boolean operators | Mental illness* OR severe mental illness*mp. OR SMI OR Schizophrenia Spectrum and Other Psychotic Disorders OR Schizophrenia mp. OR bipolar dis*mp. | Health che*mp. OR annual health che*. |
| Limits | English Language  Humans only  All adult (19+years)  01.01.2004-30.01.2025 | English Language  Humans only  All adult (19+years)  01.01.2004-30.01.2025 |

EMBASE search strategy

| Boolean operators | **1** | AND  2 |
| --- | --- | --- |
| Search Terms and Boolean operators | Mental illness* OR severe mental illness*mp. OR SMI OR Schizophrenia Spectrum and Other Psychotic Disorders OR Schizophrenia mp. OR bipolar dis*mp. | Health che*mp. OR annual health che*. |
| Limits | English Language  Humans only  All adult (19+years)  01.01.2004-30.01.2025  Remove MEDLINE records | English Language  Humans only  All adult (19+years)  01.01.2004-30.01.2025  Remove MEDLINE records |

PsycINFO Search Strategy

| Boolean operators | **1** | AND  2 |
| --- | --- | --- |
| Search Terms and Boolean operators | Mental illness* OR severe mental illness*mp. OR SMI OR Schizophrenia Spectrum and Other Psychotic Disorders OR Schizophrenia mp. OR bipolar dis*mp. | Health che*mp. OR annual health che*. |
| Limits | English Language  Humans only  All adult (19+years)  01.01.2004-30.01.2025  Remove MEDLINE records  All Journals | English Language  Humans only  All adult (19+years)  01.01.2004-30.01.2025  Remove MEDLINE records  All Journals |

CINAHL Search Strategy

| Boolean operators | **1** | AND  2 |
| --- | --- | --- |
| Search Terms and Boolean operators | Mental illness* OR severe mental illness*mp. OR SMI OR Schizophrenia Spectrum and Other Psychotic Disorders OR Schizophrenia mp. OR bipolar dis*mp. | Health che*mp. OR annual health che*. |
| Limits | English Language  Humans only  All adult (19+years)  01.01.2004-30.01.2025  Remove MEDLINE records  All Journals | English Language  Humans only  All adult (19+years)  01.01.2004-30.01.2025  Remove MEDLINE records  All Journals |

ASSIA Search Strategy

|  | **1** | Boolean operator AND  2 |
| --- | --- | --- |
| Search Terms | Psychosis  Severe mental illness  Schizophrenia  Bipolar disorder | Health checks |
| Limits | English Language  01.01.2004-30.01.2025  Dissertations and theses  Scholarly Journals  Working Papers  Articles, reports, dissertation and theses, evidence based healthcare  UK only | English Language  01.01.2004-30.01.2025  Dissertations and theses  Scholarly Journals  Working Papers  Articles, reports, dissertation and theses, evidence based healthcare  UK only |
| Additional filters | NOT (UK Biobank Ltd AND American Psychiatric Association AND Food & Drug Administration--FDA AND United Nations--UN AND Congress AND Monash University AND University of New South Wales AND University of Oslo AND University of Western Australia AND Aarhus University AND Addis Ababa University AND Congress-US AND Plymouth University AND Skype Technologies SA AND University of Barcelona AND University of Bergen AND University of Hong Kong AND University of Toronto AND University of Zurich AND WhatsApp Inc AND 23andMe Inc AND American Annals of the Deaf AND American Journal of Psychiatry AND American Psychological Association AND AstraZeneca AND Aurum Institute AND Automattic Inc AND Beijing University AND Bond University AND British Association for Psychopharmacology AND Chinese Academy of Social Sciences AND Columbia University AND Cooperative Group AND Council of Europe AND Department of Justice AND Duke University Press AND EBSCO Industries Inc AND Edith Cowan University AND Envigo AND European Commission AND European Parliament AND European Union AND Franklin University AND German Research Foundation AND Glastonbury Festival AND GlaxoSmithKline PLC AND Global Commission on Drug Policy AND GW Pharmaceuticals AND Harvard University Press AND Haukeland University Hospital AND Health Canada AND Health Research Group AND Indiana University School of Medicine AND International Bank for Reconstruction & Development--World Bank AND International Diabetes Federation AND Ipsos MORI)  NOT (SciTech Premium Collection AND Natural Science Collection AND Biological Science Collection AND Biological Science Index AND Consumer Health Database AND Biological Science Database AND Coronavirus Research Database AND Agricultural & Environmental Science Collection AND Environmental Science Collection AND Environmental Science Index AND Technology Collection AND Advanced Technologies & Computer Science Collection AND Materials Science & Engineering Collection AND Engineering Collection AND Advanced Technologies & Aerospace Database AND Environmental Science Database AND Engineering Database AND Earth, Atmospheric & Aquatic Science Collection AND Agricultural Science Collection AND Engineering Index AND Agriculture Science Database AND Materials Science Collection AND Science Database AND Healthcare Administration Database AND Meteorological & Geoastrophysical Abstracts AND Materials Science Database AND ABI/INFORM Collection AND ABI/INFORM Global AND Criminology Collection AND Criminal Justice Database AND Career & Technical Education Database AND Career & Technical Education Database: Health & Medicine AND Politics Collection AND PAIS Index AND Research Library: Literature & Language AND Research Library: The Arts AND Research Library: History AND UK & Ireland Database AND Political Science Database AND Sports Medicine & Education Index AND Earth, Atmospheric & Aquatic Science Database AND Art, Design & Architecture Collection AND Education Research Index AND Library & Information Science Collection AND Advanced Technologies & Aerospace Index AND Library Science Database AND Library & Information Science Abstracts (LISA) AND Asian & European Business Collection AND ProQuest One Literature AND Computer Science Database AND Australia & New Zealand Database AND ASFA: Aquatic Sciences and Fisheries Abstracts AND East & South Asia Database AND Middle East & Africa Database AND ABI/INFORM Trade & Industry AND Aquatic Science & Fisheries Abstracts (ASFA) 1: Biological Sciences & Living Resources) | NOT (UK Biobank Ltd AND American Psychiatric Association AND Food & Drug Administration--FDA AND United Nations--UN AND Congress AND Monash University AND University of New South Wales AND University of Oslo AND University of Western Australia AND Aarhus University AND Addis Ababa University AND Congress-US AND Plymouth University AND Skype Technologies SA AND University of Barcelona AND University of Bergen AND University of Hong Kong AND University of Toronto AND University of Zurich AND WhatsApp Inc AND 23andMe Inc AND American Annals of the Deaf AND American Journal of Psychiatry AND American Psychological Association AND AstraZeneca AND Aurum Institute AND Automattic Inc AND Beijing University AND Bond University AND British Association for Psychopharmacology AND Chinese Academy of Social Sciences AND Columbia University AND Cooperative Group AND Council of Europe AND Department of Justice AND Duke University Press AND EBSCO Industries Inc AND Edith Cowan University AND Envigo AND European Commission AND European Parliament AND European Union AND Franklin University AND German Research Foundation AND Glastonbury Festival AND GlaxoSmithKline PLC AND Global Commission on Drug Policy AND GW Pharmaceuticals AND Harvard University Press AND Haukeland University Hospital AND Health Canada AND Health Research Group AND Indiana University School of Medicine AND International Bank for Reconstruction & Development--World Bank AND International Diabetes Federation AND Ipsos MORI)  NOT (SciTech Premium  Collection AND Natural Science Collection AND Biological Science Collection AND Biological Science Index AND Consumer Health Database AND Biological Science Database AND Coronavirus Research Database AND Agricultural & Environmental Science Collection AND Environmental Science Collection AND Environmental Science Index AND Technology Collection AND Advanced Technologies & Computer Science Collection AND Materials Science & Engineering Collection AND Engineering Collection AND Advanced Technologies & Aerospace Database AND Environmental Science Database AND Engineering Database AND Earth, Atmospheric & Aquatic Science Collection AND Agricultural Science Collection AND Engineering Index AND Agriculture Science Database AND Materials Science Collection AND Science Database AND Healthcare Administration Database AND Meteorological & Geoastrophysical Abstracts AND Materials Science Database AND ABI/INFORM Collection AND ABI/INFORM Global AND Criminology Collection AND Criminal Justice Database AND Career & Technical Education Database AND Career & Technical Education Database: Health & Medicine AND Politics Collection AND PAIS Index AND Research Library: Literature & Language AND Research Library: The Arts AND Research Library: History AND UK & Ireland Database AND Political Science Database AND Sports Medicine & Education Index AND Earth, Atmospheric & Aquatic Science Database AND Art, Design & Architecture Collection AND Education Research Index AND Library & Information Science Collection AND Advanced Technologies & Aerospace Index AND Library Science Database AND Library & Information Science Abstracts (LISA) AND Asian & European Business Collection AND ProQuest One Literature AND Computer Science Database AND Australia & New Zealand Database AND ASFA: Aquatic Sciences and Fisheries Abstracts AND East & South Asia Database AND Middle East & Africa Database AND ABI/INFORM Trade & Industry AND Aquatic Science & Fisheries Abstracts (ASFA) 1: Biological Sciences & Living Resources) |

Google and Google Scholar search strategy

| Boolean operators |  | AND OR |
| --- | --- | --- |
| Search Terms and Boolean operators | Access to health checks  Annual Health Checks  Psychosis  Schizophrenia  bipolar disorder | AND SMI OR Severe Mental Illness  AND Severe Mental Illness  AND health checks  AND health checks  AND health checks |
| Limits | Dates searched: 1.01.2004-30.01.2025  Population: Adults > 18 years of age  Geographical area: UK only | Dates searched: 1.01.2004-30.01.2025  Population: Adults > 18 years of age  Geographical area: UK only |
