# Supplementary material for: Access, inequalities and annual health checks (AHCs) for adults living with severe mental illness in the UK: a mixed-methods systematic review
Source: BMJ Open. 2025 Aug 4;15(8):e093426. doi: 10.1136/bmjopen-2024-093426 (PMC12323543; doi:10.1136/bmjopen-2024-093426)
Supplement: online supplemental file 3 [file bmjopen-15-8-s003.docx]

**Supplemental Table 3**. Characteristics of the 35 studies

| **Author/date**  **Peer Reviewed- P**  **Grey Lit. -G** | **UK Country** | **Research Design and method of data collection** | **Healthcare setting** | **Sample size** | **%F** | **Age range** | **SMI diagnosis** | **Comparison group** | **Co-morbidities** | **Accommodation** | **Ethnicity or race** | **Person**  **seen** | **Health**  **checks** | **Barriers/**  **Facilitators to access** | **intervention** |
| --- | --- | --- | --- | --- | --- | --- | --- | --- | --- | --- | --- | --- | --- | --- | --- |
| Barnes et al.^62^  P | England | Audit  Clinical records  Quality improvement using questionnaire | Primary care | 1966 | NR | 16-66+ | Schizophrenia | No | 1.5% substance misuse, 6% Diabetes, 6% dyslipidaemia 6% hypertension. | Community Dwelling | NR | Assertive outreach teams | Metabolic Screening | Poor communication between primary and secondary care. Unreliable systems for sharing results. Not enough time to do checks and lack of basic equipment to carry out screening. | N/A |
| Beecroft et al.^41^  P | England | Cross-sectional  Structured interviews | Primary and secondary care | 309 | 18.12 | NR | psychosis | No | none identified | Community dwelling | NR | GP | Physical health checks | People in regular contact with their GP, not just attending for AHC, are less likely to have additional health problems (only 1.3% of those registered with a GP had unmet physical needs compared with  15.4% of those with no GP [*p*=0.056]). Built relationship of trust. | N/A |
| Bosanquet ^86^  G | England | Mixed methods  PhD thesis | Primary care | 32178  19 Qual.  32159 Quant. | 42.19 Qual.  50.7 Quant | 21-71 | Schizophrenia, bipolar disorder | No | NR | NR | NR | GP | Access to health checks more regular in practices with higher number of patients with SMI | Time constraints for AHC appointments. Poor communication between primary and secondary care. QOF focused attention on disease risk and recording at the expense of a person-centred approach. Older patients were more likely to attend compared to younger. | N/A |
| Butler et al.^79^  P | England | Qualitative  Interviews  (no depth) | Primary care | 14 | 28.57 | 19 to 66 | SMI, no identification | N/A | None identified | Community dwelling | NR | GP | Screening | Pts failed to identify the link between their mental and physical health. This meant that they ignored screening appointments. | N/A |
| Cockburn et al. ^88^  P | Scotland | Cross sectional  Data collected from patients on 1 day | Secondary care | 57 | 37. | 22-88 | Schizophrenia, schizoaffective disorder, bipolar disorder | No | Diabetes, cardiovascular disease, hypertension | In-patient | NR | Nurses on unit | Physical health screening | Structural barriers to accessing screening in the community.  Patients were not always able to comply with requests for screening because of their mental health.  Patients reluctant to engage in future care or changes to their behaviour – such as smoking cessation or attendance at screening. No reason given. | N/A |
| Crabb et al. ^38^  P | Scotland | Audit  Case notes | Primary care | 90 | NR | NR | SMI, no identification | No | None identified | Community dwelling | NR | 38% junior hospital Dr on admission as in-patient. 13% psychiatrist.  4% GP. | Physical health checks | Quality of physical health screening variable.  Bureaucracy and poor communication between primary and secondary care. | N/A |
| Crawford et al. ^53^  P | UK | Cross sectional  Data collected  from the National Audit of Psychosis in England and Wales | Secondary care | 5091 | 35.1 | 18-93 | Schizophrenia | No | NR | Community dwelling | 3973 (79.7%) White, 415 (8.3%) Asian/British Asian, 431 (8.6%) Black/Black British and 169 (3.4%) were mixed or from other minority ethnic communities | Psychiatrists and secondary care teams | Health checks | No access barriers mentioned.  QOF indicators used for AHCs. | N/A |
| Crawford et al. ^54^  P | England & Wales | Cross sectional longitudinal  Analysis of routinely collected data 2011, 2013, 2017 | Secondary care | 17947 | 34.56 | 18->65 | Schizophenia  Schizoaffective psychosis | No | NR | Community dwelling | White 14081 (78.46)  Asian/Asian British1399 (7.80)  Black/Black British 1533 (8.54)  Chinese or other 285 (1.59)  Mixed 374 (2.08)  Not stated 275 (1.53) | Secondary care consultants/intervention teams | Whole study focused on health checks in secondary care using QOF indicators | Although CQUIN and introduction of QOF incentives appeared positive, there was no evidence of significant improvements in the quality of other aspects of care received by people with psychosis during this period. Some evidence of falling standards of care. Suggestion is that attending to targets contributed to a reduction in aspects of care that were non-incentivised | N/A |
| Garriga et al. ^47^  P | England | Cross-sectional  Collected 2013-2017 database of people registered with general  practitioners in England (QResearch) | Primary care | 65 490 | 67.5 | 40 to 70 | SMI, no identification | Yes | diabetes, hypertension or CVD | Community dwelling | White 56409, Indian 797, Pakistani 715, Bangladeshi 520, Other Asian 524, Caribbean 841, Black African 849, Chinese 141, Other 1626, ethnicity not recorded 3608 | GP | Health checks | People with SMI or on medication were 5–10% more likely to access NHS Health Checks than people without. Having a diagnosis of diabetes, CVD or hypertension meant they were also more likely to attend AHCs. | N/A |
| Gonzalez et al. ^60^  P | England | Audit  Patient clinical records | Secondary care | N=232  Audit 1=126  Audit 2= 106 | A1=40.5  A2= 44.3 | Mean A1, 42.43s.d. 13.09. Mean A 2, 42.4  s.d.  11.86. | SMI, schizophrenia | No | None identified | Community dwelling | White British 78, Black African British 49 | Junior doctors | Health checks | Barriers were lack of staff motivation to implement health checks. Uncertainty of procedure and inadequate training of staff. Poor communication with primary care. Lack of adequate reporting systems | N/A |
| Hamilton et al.^65^  P | Scotland | Service evaluation  Routinely collected data | Primary care | 78 | 50 | 41-63 | SMI, schizophrenia, bipolar disorder | No | None identified | Community dwelling | NR | GP | AHC | Confusion as to where responsibility for screening lay-Negotiation sensitive.  Heavy staff workloads prevented screening.  CMHT cc’d into invitation for AHC and asked to support individual to attend and arrange transport. | Setting up specialised annual health check clinics for people living with SMI.  No formal evaluation. |
| Hardy & Gray ^71^  P | England | Audit  Records of patients with SMI or diabetes offered an appointment by letter between 1 January 2010 and 31 December  2010. | Primary care | 112 | 43 | 17-45+ | SMI, no identification | Yes | diabetes | Community dwelling | NR | GP | Health checks | Offering a set time and date assisted and patients with SMI responded to an additional invitation after nonattendance. Giving details about who would be doing the health check rather than asking person to make an appointment with an unnamed person also improved attendance. Offering another set appointment if one had been failed also helped. | N/A |
| Hardy et al. ^64^  P | England | Audit  Patient records September 2009 and August 2010. Patients identified from the SMI register of each of the 5 participating practices. | Primary care | 386 | 47 | 26-55 | SMI, no identification | No | diabetes | Community dwelling | NR | GP | Health screening | Primary-care clinicians believed that patients with SMI would not attend for a health check. | Training nurses in primary care to increase screening for risk factors and the level of lifestyle advice given.  No formal evaluation. |
| Hardy ^42^  P | England | Service evaluation  Evaluation forms, questionnaires, extraction from a template in patients’ computer records. Descriptive interviews. | Primary care | 34 staff members | NR | NR | SMI, no identification | No | NR | Community dwelling | NR | GP and care co-ordinators | AHCs | The work wanted to address reducing system inequalities in inviting people to AHCs. Staff failed to engage with the evaluation. It emphasises how difficult it is to implement a new service and evaluate it successfully. | Train the trainer programme. Training everyone involved in care of people living with SMI in how to increase uptake to AHCs and then how to follow through on guidance for further referrals.  No formal evaluation. |
| Hippisley-Cox et al. ^69^  P | England | Cross-sectional  Data collected from QRESEARCH database | Primary care | 701 | 52.7 | <25->85 | schizophrenia, bi-polar disorder | Yes | coronary heart disease, diabetes, stroke | Community dwelling | NR | GP | Health checks | People excluded not registered with a GP, in prison or in another long-stay institution. One size fits all approach to care | N/A |
| Howkins et al. ^39^  P | England | Pilot audit  Anonymized patient data gathered by first searching ECLIPSE  LIVE. Practices then approached and data requested for timeframes and dates. | Primary care | 9 | NR | NR | SMI, no identification | No | Learning disability | NR | NR | GP | Health checks | Lack of understanding about monitoring. Poor electronic coding. Lack of AHC completion. Lack of understanding around capacity and consent. | N/A |
| Joury et al.^43^ | England | Qualitative  Quality improvement study  Online questionnaires, face-to-face interviews and focus groups | Primary and Secondary care | 22 | NR | NR | SMI, no identification | No | NR | NR | NR | GP and nurses | Health checks | Provided examples of best practice. No national financial model to incentivise physical health checks beyond the six core components.  Excluded experts by experience. Didn’t have a whole team approach to ensure access is realised. | N/A |
| Kerrison et al. ^48^  P | England | Cross-sectional  Researchers used the Clinical  Practice Research Datalink (CPRD) Aurum dataset. | Primary care | 69641 | 52.91 | 25-74 | Schizophrenia, bi-polar disorder, psychosis | Yes | NR | Community dwelling | 71.28 White, 6.99 Asian, 6.60 Black, 1.83 Mixed, 0.77 Other, 12.47 Unknown | GP | Screening programmes | Screening participation was lower among adults living with SMI, than without, for bowel (42.11% vs. 58.89%), breast (48.33%  vs. 60.44%) and cervical screening (64.15% vs. 69.72%; all p < 0.001). Participation was lowest in those with schizophrenia (bowel,  breast, cervical: 33.50%, 42.02%, 54.88%), then other psychoses (41.97%, 45.57%, 61.98%), then bipolar disorder (49.94%, 54.35%,  69.69%; all p-values < 0.001, except cervical screening in bipolar disorder; p-value > 0.05). Participation was lowest among people living with SMI who live in the most deprived quintile of areas (bowel, breast, cervical: 36.17%, 40.23%, 61.47%), or are of a Black ethnicity (34.68%, 38.68%, 64.80%). Higher levels of deprivation and diversity, associated with SMI, did not explain the lower participation in screening.  Lack of support to access services. | N/A |
| Kontopantelis et al. ^44^  P | UK | Cross-sectional/ Longitudinal  CPRD computerised database of anonymised primary care medical records. | Primary care | 346 551 | NR | 18->61 | schizophrenia; affective psychoses (bipolar disorder or other unspecified affective psychosis); other types of psychosis | Yes | hypertension, asthma, hypothyroidism, osteoarthritis, kidney disease, CVD, epilepsy, COPD, cancer, stroke, heart failure, rheumatoid arthritis, dementia and psoriasis | Community Dwelling | NR | GP | QOF health check | People living with SMI appeared less healthy post-QOF. Consultation rates are higher if need is translated into greater service utilisation. | N/A |
| Launders et al.^55^  P | UK | Cross-sectional  Clinical Practice Research Datalink (CPRD) GOLD and  Aurum databases to collect de-identified data | Primary care | 216136 | 48.3 | 26-84 | Schizophrenia, bipolar disorder, psychosis | No | CVD | Community dwelling | Asian 3.6,  Black 4.6,  Mixed race 1.3  Other 1.9  White 51.2  Missing 37.4 | GP | AHCs | Screening reduced 2014-2018 with the removal of the QOF incentives.  Poor co-ordination between primary and secondary care  People living with SMI are not reliably receiving invitations for regular comprehensive physical health checks in primary care. | N/A |
| Lister et al.^45^  G | UK | Mixed Methods  Clinical Practice Research Datalink (GOLD). Hospital events Statistics [HES] database.  Telephone and F2F interviews. | Primary care | N=14857 (39 in Qual, 14838 in Quant) | Quant: 51.90 | 18-60 | Schizophrenia, schizoaffective disorder, bipolar disorder, psychosis | Yes | Diabetes, CVD, hypertension, LD, dementia, | Community dwelling | Quant: White 84.3, Asian 3.45, Black 3.18, Mixed 1.16, Other 1.44, Unknown 6.49 | HCPs | Health checks | Physical health not recognised by staff. Physical and mental health problems often treated in separate services, created confusion about responsibility.  Multi-morbidity and mood changes made access to health challenging. | N/A |
| Matias et al. ^56^  P | England | Cross sectional  UK primary  care data from the Clinical Practice  Research Datalink between April 2011  and March 2020 | Primary care | 8700 | NR | >18 | schizophrenia; affective psychoses (bipolar disorder or other unspecified affective psychosis); other types of psychosis | No | Asthma, atrial fibrillation, cancer, CVD, chronic kidney disease, chronic liver disease and viral hepatitis, COPD, diabetes, hypothyroidism, rheumatoid arthritis, stroke and TIA | Community dwelling | White, black and other minorities | GPs | Screening | QOF incentives positively affect the uptake of physical health checks for people living with SMI | N/A |
| Panesar ^87^  P | England | Audit  Data collected during screening appointments | Secondary care | 60 | 23.33 | 20-66 | SMI no identification | No | obesity | Residential rehabilitation unit | NR | Psychiatrist | Health screening | No readily available protocols or guidelines for routine physical health screening. Little evidence of routine health care interventions. | N/A |
| Pearsall et al. ^70^  P | Scotland | Qualitative (GT)  Interviews about experiences after having a health check and referral to another health programme | Primary care | 13 | 50 | Mean 54.6 | SMI, no identification | No | NR | Community dwelling | NR | GP | Health check | Initial contact with the service user by mailed letter achieved a poor response. Participation seemed to be better if the approach first made by the service user’s own community keyworker. Current level of mental health affected by individual ability to access services. | N/A |
| Pinto et al. ^52^  P | England | Cross-sectional  Lambeth DataNet; database of computerized general practice  case records derived from practices in an inner city London borough | Primary care | 903 | NR | 16-74 | schizophrenia, bi-polar, psychosis | No | hypertension, diabetes, raised cholesterol | Community dwelling | 501 White, 403 Black, 57 Mixed race, 51 Asian, 35 Chinese/Other | NR | Screening | Discriminatory approaches to black people | N/A |
| Pitman et al. ^51^  P | England | Cross-sectional  Forced-choice survey questionnaires | Primary care | 227 | 47 | mean 44.2, s. d. 12.3 | 39% Schizophrenia, 17% Bi-Polar, 14% psychosis, 20% depression, 5% unknown | No | Cardiovascular disease | Community dwelling | 139 White, 30 Black, 13 Asian, 2 Chinese, 43 unknown | Primary care and Community Mental Health Team staff | Health checks | Lack of culturally competent, inclusive and integrated care. | N/A |
| Reilly et al. ^46^  P | England | Cross sectional  data from  electronic patient secondary mental healthcare and primary care  medical records | Primary and Secondary care | 297 | 43.77 | Mean 47 | schizophrenia, bi-polar disorder, psychosis | Yes | diabetes, asthma, chronic obstructive pulmonary disorder, epilepsy, hypertension, stroke, thyroid disorder, ischaemic heart disease, heart failure, chronic  kidney disease, learning disability, hearing problems, rheumatoid arthritis, cancer, osteoarthritis, obesity, visual problems | Community dwelling-36% lived alone | 33% Black  and minority ethnic groups. 15% Asian, 12% Black and 6% mixed  ethnicity. quarter of ethnicity data missing. | GPs and CMHT | AHCs | Women’s health checks were lower than the general population, only 24% of eligible patients referred for cervical screening.  Lack of integrated information systems means that primary care is largely unaware of the extensive input from secondary mental healthcare. Poor continuity of care. | N/A |
| Ride et al. ^61^  P | England | Cross sectional Longitudinal  Documentary analysis of CPRD data | Primary and Secondary care | 5158 | 53 | 18->65 | schizophrenia, bi-polar disorder, psychosis | No | high cholesterol, hypertension, diabetes, asthma, osteopenia/osteoporosis and fatty liver disease | Community dwelling | NR | NR | Health review | Poor communication between primary and secondary care. | N/A |
| Roberts & Mwebe ^49^  P | England | Audit  Electronic patient records | Primary care | 54 | 56 | 18-88 | schizophrenia, psychosis, bi-polar | No | high cholesterol, hypertension, diabetes, asthma, osteopenia/osteoporosis and fatty liver disease | Community dwelling | NR | NR | Health checks | Poor communication between primary and secondary care. DNAs failed to be followed up. Call-recall systems needed in primary care to tackle non-attendance. | N/A |
| Roberts et al. ^85^  P | England | Audit  Case matched patient records retrieved | Primary care | 195 | 41.53 | mean 42.8, s.d.9.87 | schizophrenia | Yes, | asthma | Community dwelling | NR | GP | Screening | People with schizophrenia less likely invited for screening than general population. | N/A |
| Shah et al. ^40^  P | England | Evaluation  PA-led enhanced physical health clinic | Primary care | 71 | 45.07 | NR | SMI, no identification | No | None identified | Community dwelling | NR | PAs | Screening | Communication between primary and secondary care challenging. | Implementation of a PA-led enhanced physical health  Clinic. PAs focused on hard to reach groups of people with SMI, carrying out home visits and building relationships. No evaluation. |
| Shaw et al.^57^  P | England | Qualitative  Semi-structured interviews | Primary care | 8 pts  + 5 staff | 62.5  80.0 | Pts  40-79  Staff  40-69 | SMI, no identification | No | Cardiovascular disease | Community dwelling | Pts- 5 white  2 Mixed white  1 African British  Staff 4 white  1 Mixed white | Peer coach | Screening. | Barriers to access were language and continuity of interpreters and nurses.  Structural barriers; time, resources, provision of support. Communication between staff challenging.  Facilitators- longer regular appointments. Building positive, trusting relationships with people living with SMI. Having a peer with lived experienced assisted because people felt validated. Flexibility promoted pt. engagement and motivation. | Primrose A- peer coach with lived experience of SMI. People living with SMI who came for health checks encouraged to set own physical and MH goals which they discussed with the peer. PH +MH tracked frequently rather than 1 AHC. Aim is for increased access and integrated PH and MH care. |
| Smith et al. ^59^  P | England & Wales | Service evaluation of nurse-led well-being programme  Data gained from patient registers and notes | Secondary care | 956 | 49 | <30->49 | SMI. No identification | No | NR | Community dwelling | 826 White, 5 White European, 19 Black African, 32 Black British, 26 Black Caribbean, 5 Mixed race, 40 South East Asian, 3 Far East Asian | Nurse | Support programme | Not every patient had a complete record of measures  despite extensive nurse training,  No exploration of people choosing not to engage.  Cardiovascular risk reduced by nurse led intervention. Patients saw same nurse frequently. No exploration of relationship. | N/A |
| Vasudev & Martindale ^50^  P | England | Audit  Patient records. | Secondary care | 142 | 23.23 | 14-35 | psychosis | No | None identified | Community dwelling | 134 White, 8 unknown | NR | AHC | Having a team champion regularly raising the matter of physical health for people with SMI assisted in increasing number of patients sent AHCs. Better liaison with primary care health services further provided staff with the confidence and encouragement to direct their patients towards physical health checks. | N/A |
| Vasudev et al. ^37^  P | England | Audit  Monitoring sheet for physical health introduced | Secondary care. Medium-secure secondary care in-patient unit | 15 | 0 | NR | SMI, no identification | No | None identified | Forensic unit | NR | GP | Health checks | Poor access to physical health care (GPs) on medium secure forensic units.  Psychiatrists felt they did not know enough about physical health to prescribe. | N/A |

**Key**: AHC-Annual Health Check; A&E-Accident and Emergency; CMHT- Community Mental Health Team; CPRD- Clinical Practice Research Datalink; COPD- Chronic Obstructive Pulmonary Disease; CVD-Cardio-vascular Disease; DNA-Did Not Attend; ECG-Electrocardiogram; GP- General Practitioner; HCP-Health Care Professional; NR-Not Recorded; PA- Physician Associate; QOF-Quality Outcomes Framework
