# Supplementary material for: Access, inequalities and annual health checks (AHCs) for adults living with severe mental illness in the UK: a mixed-methods systematic review
Source: BMJ Open. 2025 Aug 4;15(8):e093426. doi: 10.1136/bmjopen-2024-093426 (PMC12323543; doi:10.1136/bmjopen-2024-093426)
Supplement: online supplemental file 5 [file bmjopen-15-8-s005.docx]

**Supplemental Table 5**. Dimensions and abilities of access using Levesque et al.^34^

| **Author / Year** | **Access focus (Dimensions VS abilities)** | **Number of Dimensions/ abilities Explored /10** | **Dimensions and abilities explored.**  **(Yes/ No)** | | | | | | | | | |
| --- | --- | --- | --- | --- | --- | --- | --- | --- | --- | --- | --- | --- |
|  |  |  | Approachability | Acceptability | Availability/  accommodation | Affordability | Appropriateness | To  perceive | To  seek | To  reach | To  pay | To  engage |
| Barnes et al.^62^ | Dimensions | 4 | Yes | Yes.  AHCs lower for older people, compared to younger. No reason given | Yes  Poor communication  Systems between primary and secondary care. | N/A | Yes.  QOF increased access initially then plateaued. | No | No | No | N/A | No |
| Beecroft et al.^41^ | Dimensions | 3 | Yes | Yes.  CMHT not as effective as GPs for monitoring physical and mental health | No | N/A | Yes. Seeing a GP regularly for AHCs enables access to care (p<0.11) | No | No | No | N/A | No |
| Bosanquet ^86^ | Both | 3 | Yes | No | No | N/A | Yes support from key workers enables access to AHCs. QOF focused attention on disease risk and recording. Poor communication between primary and secondary care. | Yes.  People aware they need AHCs | No | No | N/A | No |
| Butler et al.^79^ | Both | 8 | Yes | Yes.  Consideration of people’s needs. | Yes. Training needs of practitioners. Lack of resources. Uncertainty of role. Poor communication  Systems between primary and secondary care. | N/A | Yes.  Poor integration of physical and mental healthcare. | Yes. Variable awareness of link between physical and mental health. People attend because AHC is offered | Yes | Yes. Mobility and transport issues for people living in rural areas. | N/A | Yes. existing therapeutic  relationship |
| Cockburn et al. ^88^ | Both | 5 | Yes | Yes.  In-person review was amended accommodating length of time a person could tolerate | Yes.  Variable provision of screening. | N/A | No | Yes.  People attend AHC but reluctant to engage with behaviour change | No | No | N/A | Yes, status of mental health can be a barrier to engaging |
| Crabb et al. ^38^ | Dimensions | 3 | Yes | No | Yes.  Poor communication  Systems between primary and secondary care.  Quality of AHC variable.  Training need for primary and secondary care practitioners | N/A | Yes.  No baseline AHC. People could not be followed and assessed. | No | No | No | N/A | No |
| Crawford et al. ^53^ | Dimensions | 4 | Yes | Yes. Discusses AHCs but documented evidence of monitoring for physical health problems falls well below agreed standards. Physical health was more likely to be monitored in those with family histories of risk factors. | Yes.  Mental health practitioners need to have the knowledge and skills to assess and treat MH and PH.  cooperation between primary and secondary care  needs improvement. | N/A | Yes.  People believe they are receiving good standard of care, because they are attending AHCs. Audit demonstrates this is not the case because many health checks not attempted | No | No | No | N/A | No |
| Crawford et al. ^54^ | Dimensions | 2 | Yes | No | No | N/A | Yes. CQIN/QOF improved access. No stats to confirm extent. | No | No | No | N/A | No |
| Garriga et al. ^47^ | Both | 3 | Yes | Yes.  Higher attendance at AHCs in people with SMI. Men more likely to attend AHC than women | No | N/A | No | No | No | Yes.  People living in the most  deprived areas (quintiles Q4 and Q5) were less likely to attend  an NHS Health Check than those in the most affluent quintile  (Q1) | N/A | No |
| Gonzalez et al.^60^ | Dimensions | 2 | Yes | No | Yes  Training needs for primary and secondary care physicians.  Poor communication  Systems between primary and secondary care. | N/A | No | No | No | No | N/A | No |
| Gutacker et al. ^58^ | Both | 3 | Yes | No | No | N/A | Yes  QOF indicators as an incentive to send appointment to screen people. | No | Yes, distance to nearest MH hospital or acute care unit. | No | N/A | No |
| Hamilton et al.^65^ | Both | 6 | Yes | Yes consideration given about ability to attend and need for support | Yes, separate rooms booked for individualised and focused health check. | N/A | Yes,  CMHT cc’d into appointment Asked to support people to ensure attendance | No | No | Yes, supported with transport provision. | N/A | Yes, supported by CMHT |
| Hardy & Gray ^71^ | Both | 4 | Yes | Yes  Consideration given to fluctuating mental health for people living with SMI. Followed up if they missed an appointment for an AHC. | No | N/A | Yes.  Letter to people with set time, date and who would do the AHC improved uptake. Followed up by a telephone call either to the person and/or to their carer or CMHT. | Yes.  People with diabetes were 2.2 times  more likely to attend an AHC compared with those with SMI (OR 52.20, 95%  CI51.13–3.62) | No | No | N/A | No |
| Hardy et al.^64^ | Dimensions | 3 | Yes | Yes  Low rates of AHC for 16-55 year olds with SMI. No explanation given. | Yes  AHC fails to screen 16-55 year olds regularly. No explanation given | N/A | No | No | No | No | N/A | No |
| Hardy^42^ | Dimensions | 3 | Yes | Yes, low rates of AHCs for people living with SMI. No explanation given | No | N/A | Yes, trying to increase integrated care and encouraging the whole team to work together to increase uptake of AHCs | No | No | No | N/A | No |
| Hippisley-Cox et al.^69^ | Dimensions | 2 | Yes | No | Yes.  People with CHD and schizophrenia are less likely to receive effective monitoring. Possibility that the differential  use of exception codes removes people with schizophrenia, masking any existing inequality | N/A | No | No | No | No | N/A | No |
| Howkins et al. ^39^ | Dimensions | 2 | Yes | No | Yes  AHC for all people with LD, but does not ensure treatment is followed through | N/A | No | No | No | No | N/A | No |
| Joury et al.^43^ | Dimensions | 3 | Yes | No | Yes, not using all members of primary care team to ensure integration of care.  Training needs for healthcare assistants (and other physical health checks staff) regarding engagement with people living with SMI and carers. | N/A | Yes, invite/remind people on the SMI register  regarding physical health checks (such as using letters and  text messages, phone calls and interpreters when needed). Dedicated mental health and call/recall leads. Regularly updated SMI registers. Lack of local engagement with people living with  SMI and their supporters to understand barriers and facilitators to accessing health checks. No sexual or oral health checks incorporated into AHC. | No | No | No | N/A | No |
| Kerrison et al. ^48^ | Dimensions | 2 | Yes | Yes  Focus is on cancer screening for people living with SMI, part of AHC for some practitioners  Discusses exclusion of trans men with SMI from invitations. | No | N/A | No | No | No | No | N/A | No |
| Kontopantelis et al. ^44^ | Dimensions | 2 | Yes | No | Yes.  QOF incentive to call people in for AHC, no evidence as to whether treatment needs followed up. | N/A | No | No | No | No | N/A | No |
| Launders et al.^55^ | Dimensions | 2 | Yes | No | No | N/A | Yes.  QOF incentive for AHCs | No | No | No | N/A | No |
| Lister et al.^45^ | Both | 4 | Yes | No | Yes,  Training needed on | N/A | Yes.  QOF as an incentive to call people in for AHC. Value of support from family and trusting relationships with healthcare providers | Yes, with support. People prioritised their mental health over physical health. | No | No | N/A | Yes, development of trusting relationships |
| Matias et al. ^56^ | Dimensions | 2 | Yes | No | No | N/A | Yes.  QOF indicators increase invitations to people to increase attendance for AHCs | No | No | No | N/A | No |
| Panesar ^87^ | Dimensions | 2 | Yes | No | No | N/A | Yes  Lack of protocols or guidelines for people living with SMI as in-patients. No evidence of health promotion as a result of a health check. | No | No | No | N/A | No |
| Pearsall et al. ^70^ | Both | 7 | Yes | Yes  Methods of contacting people changed and key worker and/or CMH team involved | Yes. Guidance required on AHCs for staff. Training needs required for all staff to integrate MH and PH | N/A | No | Yes | Yes | Yes | N/A | Yes. Fluctuating mental health barrier to accessing services |
| Pinto et al. ^52^ | Dimensions | 3 | Yes | Yes | No | N/A | Yes  People sent appointments for regular checks related to QOF. No other form of checks carried out. | No | No | No | NA | No |
| Pitman et al.^51^ | Both | 6 | Yes. | Yes.  CMHT staff held more optimistic views than primary care staff about service users’ engagement.  Little regard for needs of different cultural or ethnic groups to increase access to AHCs | No | N/A | Yes.  CMHT discussed different aspects of health with people living with SMI and reasons for AHCs. Primary care team focused on assessing risk at AHCs. Structured interventions created a barrier. | Yes.  Knowledge of clinics. | No | Yes.  Travelling to appointments a barrier. Time involved in attending clinics a barrier. | N/A | Yes. Resistance to referral for treatment. Factors on how to support people not discussed. |
| Reilly et al. ^46^ | Both | 3 | Yes | No | No | N/A | Yes. QOF checks in records. Lack of integrated information systems acted as a barrier to continuity of and connected care.  Support from practice nurse or support worker enabled engagement | Yes.  Variations though with more clinics in affluent areas. | No | No | N/A | No |
| Ride et al. ^61^ | Dimensions | 2 | Yes | No | No | N/A | Yes.  QOF indicators used at AHC. | No | No | No | N/A | No |
| Roberts & Mwebe ^49^ | Dimensions | 2 | Yes | No | No | N/A | Yes.  QOF indicators used for AHCs.  DNA appointments for AHC not followed up. | No | No | No | N/A | No |
| Roberts et al.^85^ | Dimensions | 2 | Yes | No | No | N/A | Yes.  QOF indicators used variably. People with schizophrenia less likely to receive an AHC (no stats). | No | No | No | N/A | No |
| Shah et al. ^40^ | Both | 5 | Yes | Yes.  People considered in terms of need and offered outreach visits. | No | N/A | Yes.  PAs undertook outreach work with home visits. Limitation: PAs new to mental health care | No | No | Yes  People identified with difficulty reaching GP reviewed by PA at home. | N/A | Yes. Fluctuating mental health made it difficult to engage |
| Shaw et al.^57^ | Both | 8 | Yes | Yes, people considered in terms of need. Fluctuating mental health meant being flexible and using regular telephone contact until people feel able to attend in person. | Yes, training needs for all staff to recognise the need for integrated care. | N/A | Yes,  Peer coach intervention in primary care.  Structural barriers to provision; time, resources | Yes, peer coach improved perceptions of health and control given over to people to decide, with support about aspects of PH and MH that needed more focus | Yes  having one person as a direct contact improved knowledge of services | Yes, fluctuating MH meant using telephone to review frequently until people able to attend in person | N/A | Yes, building trusting relationships enabled engagement.  Ability to communicate because of language barriers an issue. |
| Smith et al.^59^ | Dimensions | 3 | Yes | Yes  Different needs of people considered. | No | N/A | Yes.  Nurse-led intervention in secondary care increased access to AHCs and health interventions. No exploration of people not engaging. | No | No | No | N/A | No |
| Vasudev & Martindale ^50^ | Dimensions | 3 | Yes | Yes  GPs not sending out appointments for people living with SMI | No | N/A | Yes.  Early intervention (EI) service used to increase AHCs. QOF indicators used. Practice nurse contacted people to enable access. Increased liaison between EI service and primary care improved access. | No | No | No | N/A | No |
| Vasudev et al. ^37^ | Both | 4 | Yes | No | Yes  Training needs around physical health checks needed for secondary care doctors | N/A | Yes  People not followed up after initial screening. | No | No | No | N/A | Yes  People refused to engage with lifestyle modifications. Reasons not explored |

Key: CMHT- Community Mental Health Team; CQUIN-Commissioning for Quality and Innovation; N/A- not applicable; QOF- Quality Outcomes framework

**Explanation of dimensions and abilities of access**

Dimensions relate mostly to the provider side of services whereas abilities relate to the person [patient] and their needs. These are not discrete and can cross-over between the different dimensions and abilities.

1. **Approachability** is related to people knowing there is a service and they can reach it, in this instance whether there was a service offering AHCs or regular screening and whether it was known to the community. Complementary to approachability is individual **ability to perceive** the need for care, in this case whether people living with SMI perceive that they need to attend AHCs to monitor their physical as well and mental health.
2. **Acceptability** is related to cultural and social factors determining the possibility for people to accept the aspects of the service, in this instance this can relate to the attitudes of medical professionals towards people living with SMI and how equitably the services are organised to take into account different characteristics. Complementary to acceptability is individual **ability to seek** care and this relates to knowledge about options regarding AHCs and the right to seek care and capacity to employ choice in order to seek care. This aims towards ensuring the AHC in health care meets the needs of different cultural, socioeconomically disadvantaged and people with SMI in this study.
3. **Availability and accommodation** relates to whether healthcare services can be reached physically and in a timely manner, in this case an AHC can identify problems and intervene before a person living with SMI reaches crisis and has to be admitted as an in-patient. Availability concerns the physical existence of health resources have sufficient capacity to produce services. This is about whether a service is urban or rural, working hours, mode of service provision (contact procedure and type of consultation), transport facilities and also qualifications of practitioners (or training) to focus on the physical and mental health needs of people living with SMI. Access becomes restricted if there is an uneven distribution of healthcare services across a country or across services, for example if secondary care is developed at the expense of primary care. Complementary to availability and accommodation is individual **ability to reach** healthcare, in this case for an AHC. This relates to mobility, availability of transport, flexibility of occupation and knowledge of healthcare services. One example is if a person living with SMI experiences a temporary chaotic existence then this inhibits their ability to reach healthcare to achieve an AHC.
4. **Affordability** is about whether a healthcare service is priced appropriately to take into account the population it serves. Currently in the UK, the NHS is free at the point of access for adults, apart from dental services. Complementary to affordability is individual **ability to pay**, for people living with SMI, prescriptions for treatment are currently free for long-term conditions and healthcare services are also free at the point of access. The only area where ability to pay may take precedent is being able to reach services and reliant on public transport or taxis.
5. **Appropriateness** relates to the fit between healthcare services and needs of people living with SMI, its timeliness, the amount of care spent in assessing health problems and determining the correct treatment and the technical and interpersonal quality of the services provided. Therefore, if an AHC fails to take a holistic approach to the physical and mental health needs of people living with SMI then it is inappropriate. Appropriateness is also about quality of the way services are provided, whether they are integrated and exhibit continuous care. One example, is the ability of people living with SMI to choose how to utilise healthcare and whether these services generate satisfaction as an outcome. Complementary to appropriateness is individual **ability to engage** in an AHC and related services. This relates to choice and decision-making, which in turn relates to capacity, or whether capacity fluctuates for some individuals, which increases the complexity of healthcare. Also linked is individual capacity to communicate as well as notions of health literacy, self-efficacy and self-management. This is in addition to the importance of receiving care that is actually appropriate for the person living with SMI.
