# Supplementary material for: Access, inequalities and annual health checks (AHCs) for adults living with severe mental illness in the UK: a mixed-methods systematic review
Source: BMJ Open. 2025 Aug 4;15(8):e093426. doi: 10.1136/bmjopen-2024-093426 (PMC12323543; doi:10.1136/bmjopen-2024-093426)
Supplement: online supplemental file 6 [file bmjopen-15-8-s006.docx]

**Supplemental Table 6**. AHCs for people with SMI using PROGRESS Plus^35^ and Core20PLUS^23^

|  | **Place of residence using IMD** | **Race, culture, ethnicity, language** | **Occupation** | **Gender, Sex (including gender identity)** | **Sexual Orientation** | **Religion** | **Education** | **Socio-economic status** | **Social Capital** | **Age** | **disability** | **Pregnancy or maternity** |
| --- | --- | --- | --- | --- | --- | --- | --- | --- | --- | --- | --- | --- |
| Barnes et al.^62^ | No | No | No | No | No | No | No | No | No | Yes,  Modest increase in metabolic screening by age | No | No |
| Beecroft et al.^41^ | No | No | No | Male or female, not mentioned further | No | No | No | Yes, but then failed to mention it in the discussion | No | Yes, but failed to indicate if any significance | No | No |
| Bosanquet ^86^ | No | No | No | Yes M or F not mentioned further | No | No | No | No | No | Yes  Younger patients less likely to attend AHCs; older patients were more likely. | No | No |
| Butler et al.^79^ | No | No | No | Male or female, not mentioned further | No | No | No | No | No | Yes, but failed to indicate if any significance | No | No |
| Cockburn et al.^88^ | No | No | No | Yes, not mentioned further | No | No | No | No | No | Yes, nothing further | No | No |
| Crabb et al.^38^ | No | No | No | No | No | No | No | No | No | No | No | No |
| Crawford et al.^53^ | No | Yes, not mentioned further | No | Yes, not mentioned further | No | No | No | No | No | Yes not mentioned further | No | No |
| Crawford et al.^54^ | No | Yes, no sig | No | Yes, not mentioned further | No | No | No | No | No | Yes, not mentioned further | No | No |
| Garriga et al.^47^ | Yes | Yes ethnicity. Not mentioned further | No | Yes, not mentioned further | No | No | Yes, not mentioned further | No | No | Yes, not mentioned further | No | No |
| Gonzalez et al. ^60^ | Yes | Yes, ethnicity, no significance | No | Yes, not mentioned further | No | No | No | Yes | No | Yes, not mentioned further | No | No |
| Gutacker et al. ^58^ | No | Yes. Non-white. Not mentioned further | No | Yes, not mentioned further | No | No | No | Yes, mentioned benefits, but did not expand further | No | Yes, not mentioned further | No | No |
| Hamilton et al. ^65^ | No | No | No | Yes, not mentioned further | No | No | No | No | No | Yes, not mentioned further | No | No |
| Hardy & Gray ^71^ | No | No | No | No | No | No | No | No | No | Yes, older people more likely to adhere to letter for a health check | No | No |
| Hardy et al.^64^ | No | No | No | No | No | No | No | No | No | Yes, older people more likely to be screened | No | No |
| Hardy^42^ | No | No | No | No | No | No | No | No | No | No | No | No |
| Hippisley-Cox et al.^69^ | Yes, schizophrenia group strongly skewed towards the most deprived  quintile | No | No | Yes, Patients  with schizophrenia and bipolar disorder more likely to be female | No | No | No | No | No | Yes, Patients  with schizophrenia more likely to be younger | No | No |
| Howkins^39^ | No | No | No | No | No | No | No | No | No | No | Intellectual disability, no increase in AHCs. | No |
| Joury et al.^43^ | No | Yes, staff displayed lack of cultural competence when dealing with pts from different cultural backgrounds | No | No | No | No | No | No | No | No | No | No |
| Kerrison et al.^48^  NHS Core  20PLUS5 initiative | Yes, participation in health checks was lowest among people living in the most deprived  quintile of areas. | Yes, Participation by adults of an ethnic minority background living with SMI  was  particularly low | No | Yes.  Trans men with a cervix and non-binary females excluded from screening invites. | No | No | No | No | No | Yes, not mentioned further | No | No |
| Kontopantelis et al.^44^ | Yes, not mentioned further | No | No | Yes, females more likely to consult than males | No | No | No | No | No | Yes. Higher number of consultation rates in older people | No | No |
| Launders et al.^55^ | No | Yes,  Patients of Black (vs White)  ethnicity were more likely to always have complete screening  in the 2014–2018 period only (OR 1.39; 95% CI 1.19 to 1.62) | No | Yes. Receiving no screening in the 2014–2018 and  2011–2014 periods higher for men, patients of ‘other’ or missing ethnicity (compared with White ethnicity). Men on QOF register more likely to receive screening compared to women. | No | No | No | No | No | Yes,  older age was associated with  lower odds of receiving no screening (OR per 10-year  increase  in age 0.86; 95% CI 0.83 to 0.90) | No | No |
| Lister et al.^45^ | Yes not mentioned further | Yes  People of South Asian ethnicity had a reduced mortality rate and increased health checks. | No | Yes  Female patients were more likely to receive health checks (except for cholesterol tests) than males. | No | No | No | No | No | Older age > physical health checks. | Yes  type of SMI affected access to health checks | No |
| Matias et al. ^56^ | Yes, not mentioned further | Yes, not mentioned further | No | Yes, not mentioned further | No | No | No | No | No | Yes, not mentioned further | Yes co-morbid disease such as diabetes increased uptake of health checks by 22% | No |
| Panesar ^87^ | No | No | No | Yes, not mentioned further | No | No | No | No | No | Yes, not mentioned further | No | No |
| Pearsall et al.^70^ | No | No | No | No | No | No | No | No | No | No | No | No |
| Pinto et al.^52^ | Yes,  Lambeth and Southwark have a higher than  average prevalence of psychosis, which is linked to  deprivation and ethnicity characteristics of the  area | Yes, White patients were more likely to be diagnosed with BPD and black patients with  Schizophrenia. | No | Yes, no significant difference between the  white and black groups in terms of gender | No | No | No | No | No | Yes, patients with psychosis were older | No | No |
| Pitman et al. ^51^ | No | Yes, descriptive | Yes  descriptive | Yes, descriptive | No | No | No | No | No | Yes, descriptive | No | No |
| Reilly et al.^46^ | No | Yes, descriptive | Only 10% of people living with SMI in employment but no discussion about how this may constitute a barrier | Yes, women living with SMI less likely to receive cervical screening and mammograms. No reason given. | No | No | No | No | No | Yes, descriptive | No | No |
| Ride et al.^61^ | Yes | Yes | No | Yes | No | No | No | No | No | Yes | No | No |
| Roberts & Mwebe^49^ | No | No | No | Yes, descriptive | No | No | No | No | No | Yes, descriptive | No | Yes,  Only 9% of women under 45 years had received pregnancy/contraception advice |
| Roberts et al.^85^ | No | No | No | Yes, descriptive |  | No | No | No | No | Yes descriptive | patients with schizophrenia less likely to receive certain clinically important health checks | No |
| Shah et al.^40^ | No | No | No | Yes, descriptive | No | No | No | No | No | No | No | No |
| Shaw et al.^57^ | No | Yes, language a barrier if no translator | No | Yes, descriptive | No | No | No | No | No | Yes, but failed to indicate if any significance | No | No |
| Smith et al.^59^ | No | Yes, descriptive | No | Yes, descriptive | No | No | No | No | No | Yes, descriptive | No | No |
| Vasudev & Martindale ^50^ | No | No, all white population | No | Yes,  Males tended to have higher rates of cardiovascular risk factors, not measured statistically. | No | No | No | No | No | Yes, descriptive, no sig diff but not measured statistically | No | No |
| Vasudev et al.^37^ | No | No | No | Male only, descriptive | No | No | No | No | No | Yes, descriptive | No | No |
