# Supplementary material for: Access, inequalities and annual health checks (AHCs) for adults living with severe mental illness in the UK: a mixed-methods systematic review
Source: BMJ Open. 2025 Aug 4;15(8):e093426. doi: 10.1136/bmjopen-2024-093426 (PMC12323543; doi:10.1136/bmjopen-2024-093426)
Supplement: Supplementary file 7 [file bmjopen-2024-093426.reviewer_comments.pdf]

PEER REVIEW HISTORY

BMJ Open publishes all reviews undertaken for accepted manuscripts. Reviewers are asked to complete a checklist review form and are provided with free text boxes to elaborate on their assessment. These free text comments are reproduced below.

ARTICLE DETAILS

Title (Provisional)

Access, inequalities and annual health checks for adults living with severe mental illness in the United Kingdom (UK): A mixed-methods systematic review.

Authors

Owens, Janine; Ravindrarajah, Rathi; Norman, Gill; Hopkin, Elinor; Shi, Chunhu; Lovell, Karina; Bee, Penny E

VERSION 1 - REVIEW

**Reviewer**	**1**
**Name**	**Davies, Philippa**
**Affiliation**	**University of Bristol, Population Health Sciences, Bristol Medical School**
**Date**	**03-Jan-2025**
**COI**	**None**

A very confusing review. Some of the methods were hard to follow and it was even harder to relate the results to the review questions and eligibility criteria (especially the outcomes). There are also some weaknesses with the search strategy. I don’t doubt that there are some interesting findings but in its current state I don’t think it can be considered to be a good quality systematic review. Some more detailed comments below.

To be compliant with the PRISMA extension, the abstract needs an explicit statement of the main objective/questions addressed by the review and should specify the eligibility criteria and methods used to assess risk of bias.

Introduction, second paragraph – ‘with evidence suggesting increased mortality of up to 25 years’. This wasn’t clear. Does it mean a reduced life expectancy of up to 25 years?

Methods:

The methods section states that the protocol registered on PROSPERO was updated to increase clarity of wording of the outcomes, however it also looks like the review questions themselves changed, not just the outcomes (and, to be honest, I found the original review questions to be clearer).

Review question: ‘What does the evidence suggest concerning the effectiveness of interventions to improve the accessibility of AHCs for people living with SMI?’. It is not clear what is being asked here. How does this question differ from the first one?

Review question: ‘What is the existing evidence on SMI and AHCs for minority groups identified in CORE10PLUS5?’. Again this is not very clear. Existing evidence on what? Improving health outcomes? Or is the question asking whether these groups are represented within the studies identified?

There is an additional review question in the original protocol which appears to have been dropped without explanation (‘What features of interventions are associated with increased use of annual health checks for people with SMI?)

PPI – why did the ‘nature of the exploration’ mean that it was not possible to involve patients? It is interesting that the discussion is very critical of the lack of involvement of patients and public in the studies identified yet the review authors state that they couldn’t do this in their own research!

Search strategy: A big concern here is that the search strategy will miss studies of bipolar patients. Only the term ‘bi-polar disorder’ is included, and not the more commonly used spelling ‘bipolar’. Nor is the MeSH term ‘Bipolar disorder/’ used. ‘psychoses’ and ‘psychotic’ are also not included, only ‘psychosis’. The plural of ‘check up’ and checkup’ is not used which may also result in missed studies (similarly only the singular ‘screening’ is used). There is also some redundancy in some of the terms used (e.g. ‘schizophrenia-like disorder’ will be picked up by ‘schizophrenia’, ‘physical health check’ will be picked up by ‘health check’). Is there a qualifier missing for column 1 (e.g. ‘.mp’ or ‘tw’)?

Eligibility criteria – I think it would have been clearer to split these up according to each question posed. Otherwise it is a bit confusing. For example, is the intervention the health check itself or is it an intervention to increase access? The ‘interventions’ description seems to suggest that it is the health check itself but this is not consistent with the first review question. The appropriate study designs would also be expected to vary for the different review questions and not all of the review questions would warrant a ‘comparator’.

Outcomes. This section was particularly confusing and I found it very difficult to understand which outcomes are relevant to each review question. Two outcome domains are described. How do these relate to the five outcomes that follow. Do they each fit into one of the domains? If so, which?

A definition of ‘equality of access’ would be helpful. Are the review authors interested in differences in equality of access within patients with SMIs (i.e. according to other patient characteristics) or whether patients with SMIs have equality of access as patients who do not have SMIs? The review questions suggest the former, but some of the results in the table seem to concern the latter.

Which review question does ‘evidence of AHCs reducing unplanned admissions’ relate to? What is the difference between ‘uptake’ and ‘accessibility’ in terms of how these outcomes are measured? Would both not be measured in the same way i.e. numbers who get a health check?

For the barriers and facilitators studies, did these have to be formally elicited from study participants (e.g. through surveys, focus groups, interviews) or have you just included the

opinions of the authors of the papers? I get the impression that it may be the latter (only two qualitative studies are included). If so, this would be highly biased?

More information in the methods on how the PROGRSS tool would be helpful. Was it used to look at who was included in the studies, or was it used to look for differences in the effects of the interventions according to PROGRESS characteristics?

How exactly were Levesque's five dimensions of accessibility applied? Were these based on the judgements of the reviewers?

Assessment of methodological quality – Personally, I think that it would have made more sense to use different tools appropriate to the study design and type(s) of question being asked. A tool was used that is intended for diverse study designs so why is a separate tool also used for cross-sectional study designs? This is not clear.

Results:

Characteristics of studies – it would be helpful here to include the number of studies reporting data for each of the review questions.

The results section is extremely difficult to relate to the review questions specified in the methods. Which review question does the subheading 'Equality of access to health checks for people living with SMI' relate to? The text for this section reads as though it is actually discussing barriers and facilitators to access. Where is the actual evidence for whether interventions to improve access are effective?

'Evidence of AHCs on unplanned admissions and ACE attendance' –Five studies are reported as having addressed this question, but only 3 are described. Did all of these studies actually provide evidence of whether AHCs reduce unplanned admissions/ACE visits? Where are the actual numerical results for these studies? They do not appear in the supplementary table column 'Hospital admissions'.

'Effectiveness of interventions to improve uptake of access'. Again – where are the actual results for the six studies? They do not appear in either the text or the table. To answer this question, studies need to have a comparative element (either a control group or a before and after measurement) but this does not seem to have been the case for some studies. For example, references 49 and 50 (Pearsall 2014 and Butler 2020) are qualitative studies. Many of the 'results' reported for these studies are not access outcomes and seem more like barriers and facilitators.

The argument for 'theoretical generalisation' of the results seems weak (and would be more appropriate in the discussion). Theoretical generalization is the use of data to construct a theory not to test it. It has no place in a discussion of the evidence for the effects of something.

'Dimensions of access and equity for people living with SMI'. Where is the data on access that supports the statements made in table 3 (column: differences in levels of access to health checks)? There are some inconsistent findings in the table. For example, reference 68 found no difference in access by age but also that older adults were less likely to attend than younger and also that older adults were more likely to attend than younger adults!!

Gender and sex (Table 3). Shouldn't this be looking for differences in access for people with SMI dependent on whether they are male or female? Breast and cervical screening are not relevant as these are not offered to men.

People in contact with the criminal justice system – the results section previously cited the Hippsley-Cox paper in relation to prison populations so why does this not appear in table 3?

Discussion – should include a summary of the main findings and a discussion of the strengths and weaknesses of the review.

Conclusion – the first sentence is not supported by the evidence in the paper. Most of the conclusion seems to be very much opinion- rather than evidence-based.

Supplementary table. The data in the outcomes column is very diverse and hard to relate back to the outcomes in the review. A lot of what is reported does not even appear to map onto the review outcomes/questions. It would be helpful to be clear which outcomes are assessed in the studies and actual numerical results should be reported rather than vague statements of differences with no indication of the size or significance of these differences.

Did the authors identify/consider this study? It looks very relevant https://pubmed.ncbi.nlm.nih.gov/20219096/

**Reviewer**	**2**
**Name**	**Coffey, Michael**
**Affiliation**	**Swansea University, School of Health and Social Care**
**Date**	**09-Jan-2025**
**COI**	**None**

Excellent paper, nicely laying out the available literature to give a very clear indication of the challenges and opportunities in annual health checks for people with severe mental illness. I valued the critical take on not only the literature but also the context in which incentives are used and the likely effects of these. It put me in mind of the Kelman and Friedman paper on Performance improvement and performance dysfunction and the distortion effects of targets.

My one note on your paper is on page 6 where you describe the study population and give two different sources for the definition used of SMI. I wonder if this could be clarified and tidied up?

VERSION 1 - AUTHOR RESPONSE

Reviewer 2	Response	Revision
Excellent paper, nicely laying out the available literature to give a very clear indication of the challenges and opportunities in annual health checks for people with severe mental illness. I valued the critical take on not only the literature but also the context in which incentives are used and the likely effects of these. It put me in mind of the Kelman and Friedman paper on Performance improvement and performance dysfunction and the distortion effects of targets.<br><br>My one note on your paper is on page 6 where you describe the study population and give two different sources for the definition used of SMI. I wonder if this could be clarified and tidied up?	Thank you for your kind reflection on the paper and the work by Kelman and Friedman, which is useful for another paper I am writing on parity of esteem.<br><br>I can see that we have cited 2 different NHS papers for the definition in the introduction and then in the section on population. We will amend to just use the primary study mentioned initially.	"We included people identified as living with SMI as defined within the (NHS) long-term plan. This defines severe mental illnesses (SMI) as a diagnosis of schizophrenia, bipolar affective disorder, and other psychoses (and other patients on lithium therapy-within the preceding six months) [1].<br>We included studies of people living with SMI plus substance abuse, provided that health checks appeared in the paper. We did not include studies focusing only on dementia, personality disorders and intellectual disability. However, we included people with intellectual disabilities and people with a diagnosis of a personality disorder living with SMI."
**Reviewer 1**	**Response**	**Revision**
To be compliant with the PRISMA extension, the abstract needs an explicit statement of the main objective/questions addressed by the review and should specify the eligibility criteria and methods used to assess risk of bias.		Done within the restrictions of 250 words. Although ROB is not possible with the type of studies in the review.

Introduction, second paragraph – 'with evidence suggesting increased mortality of up to 25 years'. This wasn't clear. Does it mean a reduced life expectancy of up to 25 years?	Our apologies, there is a word missing here, it should read increased mortality rates. Happy to use the phrase you suggest though.	"with evidence suggesting a reduced life expectancy of up to 25 years compared with the rest of the population"
Review question: 'What does the evidence suggest concerning the effectiveness of interventions to improve the accessibility of AHCs for people living with SMI?'. It is not clear what is being asked here. How does this question differ from the first one?	After reading the extended comments and thoughts on questions, we have clarified and simplified them and they now align with the protocol in PROSPERO as you intimated.	What are the barriers and facilitators of access to AHCs for people living with SMI?<br><br>What features, actions, interventions or models of service provision improve uptake and access to AHCs for people living with SMI?<br><br>To what degree does the evidence on SMI and access to AHCs represent minority groups as identified in Core20PLUS?<br><br>What are the barriers and enablers of access to AHCs for people living with SMI from a minority group, as identified in Core20PLUS?
Review question: 'What is the existing evidence on SMI and AHCs for minority groups identified in CORE10PLUS5?' Again this is not very clear. Existing evidence on what? Improving health outcomes? Or is the question asking whether these groups are represented within the studies identified?	Yes, we can see that one word is missing here and it should read access to AHCs. This then asks whether these groups are represented in the studies. We have clarified the question.	To what degree does the evidence on SMI and access to AHCs represent minority groups as identified in Core20PLUS?
There is an additional review question in the original protocol which appears to have been dropped without explanation ('What features of interventions are associated with increased use of annual health checks for people with SMI?)	This is an oversight and we have amended the questions	What features, actions, interventions or models of service provision improve uptake and access to AHCs for people living with SMI?
PPI – why did the 'nature of the exploration' mean that it was not possible to involve patients?	We have clarified our statement about the nature of the exploration.	"This was a systematic review of access to annual health checks for

		people living with severe mental illness. There was minimal funding and tight time scales. This meant it was not appropriate to ask a diversity of people with lived experience to employ their time for no recompense, there was little time to develop individuals to gain insight into research methods, or critically appraise existing evidence, there was no funding or time to develop a diverse group and the relationships of trust required for effective working. Therefore, it was not possible to involve people with lived experience in the design, conduct, reporting or dissemination of this study."
Search strategy: A big concern here is that the search strategy will miss studies of bipolar patients. Only the term 'bi-polar disorder' is included, and not the more commonly used spelling 'bipolar'. Nor is the MeSH term 'Bipolar disorder/' used. 'psychoses' and 'psychotic' are also not included, only 'psychosis'. The plural of 'check up' and checkup' is not used which may also result in missed studies (similarly only the singular 'screening' is used). There is also some redundancy in some of the terms used (e.g. 'schizophrenia-like disorder' will be picked up by 'schizophrenia', 'physical health check' will be picked up by 'health check'). Is there a qualifier missing for column 1 (e.g. '.mp' or 'tw')?	Bi-polar disorder is a typographical error, thank you for picking it up.	We were concerned about your comments. Therefore, we ran the whole search again using exploded MeSH terms and have submitted an amended supplementary table of terms. This also gives a more up-to-date review because the paper was originally submitted at the beginning of August 2024, but the journal had difficulty finding reviewers.[mp added to the search terms]
is the intervention the health check itself or is it an intervention to increase access? The 'interventions' description seems to suggest that it is the	The intervention should increase access to the health check.	Section clarified: We included studies of AHCs, short health screening interventions, health

health check itself but this is not consistent with the first review question.		promotion interventions, or reasonable adjustments, which considered or improved uptake and/or access to screening for people living with SMI. We excluded studies of health checks focusing entirely on a particular physical illness (e.g. an annual asthma or diabetes review) or reviews only of the primary diagnosed SMI (e.g. annual reviews of medication for schizophrenia or bipolar disorder), unless they discussed other aspects related to increasing access to a health check. Checks took place in any healthcare setting or in the community.
A definition of 'equality of access' would be helpful. Are the review authors interested in differences in equality of access within patients with SMIs (i.e. according to other patient characteristics) or whether patients with SMIs have equality of access as patients who do not have SMIs? The review questions suggest the former, but some of the results in the table seem to concern the latter.	We are looking at equality of access to health checks for people living with SMI and belonging to a marginalised group, compared to people living with SMI and not belonging to another marginalised group.	Supplementary Table. Characteristics of the 35 studies amended to reflect the review questions.<br><br>Also added a supplementary table on Levesque's dimensions of access.
Which review question does 'evidence of AHCs reducing unplanned admissions' relate to?<br><br>What is the difference between 'uptake' and 'accessibility' in terms of how these outcomes are measured? Would both not be measured in the same way i.e. numbers who get a health check?<br>For the barriers and facilitators studies, did these have to be formally elicited from study participants (e.g. through surveys, focus groups, interviews) or have you just included the opinions of the authors of the	It does not so removed.<br><br><br>Accessibility using Levesque's definition is not merely measuring how many attend. Accessibility contains five dimensions: approachability, acceptability, availability/accommodation, affordability, appropriateness, and 5	Evidence of AHCs reducing unplanned admissions removed. To increase clarity, we have attached a supplementary table on and explanations of the dimensions and abilities of access. We used this to determine access in the paper. We did not cite the opinions of authors, merely used the

papers? I get the impression that it may be the latter (only two qualitative studies are included). If so, this would be highly biased?	abilities: ability to perceive, seek, reach, pay and engage. Barriers and facilitators of access are discussed in the papers by the authors. They are not necessarily formally elicited from participants.	dimensions and abilities of access to tease out any evidence of these facets in the papers.
More information in the methods on how the PROGRSS tool would be helpful. Was it used to look at who was included in the studies, or was it used to look for differences in the effects of the interventions according to PROGRESS characteristics?	Thank you for your interest. PROGRESS and PROGRESS PLUS are merely ways of applying an equity lens for interventions/studies. We discussed this earlier in the paper and used the framework for identifying the characteristics of participants in studies, this was blended with the protected characteristics in the Equalities Act 2010, which Core20PLUS5 focuses on.	A PROGRESS and Core20PLUS table is attached as a supplementary table to demonstrate equity in studies. This is so you can see our thinking.
How exactly were Levesque's five dimensions of accessibility applied? Were these based on the judgements of the reviewers?	No, we used Levesque's dimensions as an a priori framework, although assessing dimensions is in itself a subjective process. We acknowledged this after the table on Leveque et al.'s framework of access: *"Access is a complex concept and even though researchers applied Levesque's dimensions as an a priori framework to the evidence, they experienced challenges because of the difficulty in categorizing certain questions or data into a specific dimension or ability of access. This is because some constructs contain similar elements and a degree of overlap occurred. Engaging in lengthy discussions ensured researchers reached the most appropriate decision and fit for the evidence."*	We have attached a supplementary table of our assessment of access using Levesque's dimensions for readers to see the process.

Personally, I think that it would have made more sense to use different tools appropriate to the study design and type(s) of question being asked. A tool was used that is intended for diverse study designs so why is a separate tool also used for cross-sectional study designs? This is not clear.	We followed JBI guidance for MM SRs. JBI admit guidance lacks clarity and are currently working on this area. We added AXIS, because this was requested by a reviewer of a different and previous mixed methods SR paper.	We have deleted the AXIS table and just left QuADS as the main assessment of quality. (Supplementary table).
Characteristics of studies – it would be helpful here to include the number of studies reporting data for each of the review questions.	This is included in Table 2; it has barriers and facilitators of access and also dimensions of access.	We have revised the supplementary table on characteristics to make it clearer. We have rewritten the sections according to the review questions and this may now answer your question.
The results section is extremely difficult to relate to the review questions specified in the methods. Which review question does the subheading 'Equality of access to health checks for people living with SMI' relate to? The text for this section reads as though it is actually discussing barriers and facilitators to access. Where is the actual evidence for whether interventions to improve access are effective?	We are in agreement here and a significant amount of work has gone into rewriting this section.	Results clarified in line with the review questions.
'Evidence of AHCs on unplanned admissions and A&E attendance' –Five studies are reported as having addressed this question, but only 3 are described. Did all of these studies actually provide evidence of whether AHCs reduce unplanned admissions/A&E visits? Where are the actual numerical results for these studies? They do not appear in the supplementary table column 'Hospital admissions'.	None of these studies provide evidence and so to increase clarity, this column has been deleted	Column deleted
'Effectiveness of interventions to improve uptake of access'. Again – where are the actual results for the six studies? They do not appear in either the text or the table. To answer this question, studies need to have a comparative element (either a control group or a before and after measurement) but this does not seem to have been the case for some studies. For example, references 49 and 50 (Pearsall 2014 and Butler 2020) are qualitative studies. Many of the 'results' reported for these studies are not access outcomes and seem more like	Interventions are mostly qualitative interventions, or service improvements. These are not subject to control groups. The studies are weak, making the evidence 'thin' at best. One study wasn't an intervention so removed from the list. No interventions possessed control groups or comparative elements and	Separate column of interventions created in characteristics table. Whole table revised for clarity and so it matches review questions.

barriers and facilitators.	this is noted in the discussion.	
The argument for 'theoretical generalisation' of the results seems weak (and would be more appropriate in the discussion). Theoretical generalization is the use of data to construct a theory not to test it. It has no place in a discussion of the evidence for the effects of something.	Ok, removed.	Removed.
Dimensions of access and equity for people living with SMI'. Where is the data on access that supports the statements made in table 3 (column: differences in levels of access to health checks)?	Not sure about this comment or what it means. This may be because we have revised the tables and added in a table on dimension and abilities of access and one on PROGRESS and Core20PLUS.	This comment may already be resolved with the addition of supplementary tables to illustrate our thinking and refinement of other tables. Table 3 removed.
Gender and sex (Table 3). Shouldn't this be looking for differences in access for people with SMI dependent on whether they are male or female? Breast and cervical screening are not relevant as these are not offered to men.	Yes, we can see your point but the label is gender and sex, not merely sex.<br><br>Breast and cervical screening are available for transgender men, and non-binary people assigned female at birth, who have a cervix.	Table 3 removed and made the text clearer
People in contact with the criminal justice system – the results section previously cited the Hippsley-Cox paper in relation to prison populations so why does this not appear in table 3?	The Hippisley-Cox et al. paper is not about populations in the secure estate. The paper offers a one line comment as to people who they perceive may be excluded, amongst others, but this is not the main focus of the paper. Nor did it set out to explore prison populations. This is why it does not appear in Table 3.	Statement removed. Table 3 removed
Discussion – should include a summary of the main findings and a discussion of the strengths and weaknesses of the review.	The journal structure asks for a separate section on strengths and limitations. Which we have already provided. Adding this again in the body of the paper, we feel, makes it unnecessarily repetitive and adds to the word count.	Clarified the discussion
Conclusion – the first sentence is not supported by the evidence in the paper.	We disagree with your interpretation of the conclusion. The evidence is	Clarified the conclusion.

Most of the conclusion seems to be very much opinion- rather than evidence-based.	in the paper for discrimination, but we have made the paper clearer	
Supplementary table. The data in the outcomes column is very diverse and hard to relate back to the outcomes in the review. A lot of what is reported does not even appear to map onto the review outcomes/questions. It would be helpful to be clear which outcomes are assessed in the studies and actual numerical results should be reported rather than vague statements of differences with no indication of the size or significance of these differences.	We agree with your observations, but the evidence was contradictory, outcomes were vague in the studies and in many cases failed to relate to the outcomes in the review. There were no levels of significance in the papers, hence our observations about the lack of robust methods. We agree about the lack of outcomes in the papers and there were numerous nebulous statements, making analysis frustrating. However, papers did discuss SMI and access to AHCs, but few discussed how this was facilitated.	Table amended to barriers and facilitators of access to increase clarity for readers.
Did the authors identify/consider this study? It looks very relevant https://pubmed.ncbi.nlm.nih.gov/20219096/	Thank you, yes, we did consider this study and discussed as a group as to its inclusion. However, we decided that it did not meet our inclusion criteria because of one description of SMI as 'other SMIs' and this included depression which was not in our criteria. We decided not to include clinical depression, although some academics may consider this to be an SMI. The study authors also failed to report what they meant by 'other SMIs' and how many people fell into that category.	Not added paper.
